# Targeting the hydrophobic pockets of FAK/PYK2 FAT domain: a highly effective inhibitory strategy suppressing tumor growth and eliminating metastasis

**DOI:** 10.1186/s12964-025-02203-1

**Published:** 2025-05-19

**Authors:** Maria Christoforou, Anna Charalambous, Dimitrios Sfakianakis, Paris Alexander Skourides

**Affiliations:** Department of Biological Sciences, University of Cyprus, P.O. Box 20537, 1678 Nicosia, Cyprus

**Keywords:** FAK, PYK2, Paxillin, Cancer, Tumor progression, Metastasis

## Abstract

**Background:**

FAK is a non-receptor tyrosine kinase and an adaptor protein commonly overexpressed in cancer. It regulates multiple tumorigenic pathways through both kinase-dependent and kinase-independent scaffolding functions and thus represents a promising therapeutic target for various cancers. Several FAK kinase inhibitors shown to be effective in preclinical studies advanced to clinical trials, however none produced objective clinical responses. These results are in part attributed to drug resistance and the inability to simultaneously target kinase-dependent and kinase-independent functions of the protein, both of which have been shown to promote tumorigenesis. This has led to the development of scaffold inhibitors that could be used as adjuvants, none of which have so far reached the clinical stage. Importantly, FAK’s closely related paralogue, PYK2, compensates for the loss of FAK thus it is also important to target both kinases. In the present study, we evaluate a novel strategy for the inhibition of kinase-dependent and kinase-independent functions of FAK and PYK2 through the expression of the FAT HP-site-specific LD2-LD4 peptide that leads to their displacement from focal adhesions.

**Methods:**

The impact of LD2-LD4 expression on FAK and PYK2 was assessed through co-immunoprecipitation experiments, Western Blot analysis and quantitative immunofluorescence. In vitro investigation of the effects of LD2-LD4 expression on tumor cell migration and proliferation was carried out using 2D migration, 3D invasion and proliferation assays. The preclinical experiments of this study were carried out using an orthotopic xenograft model, followed by immunohistochemical analysis.

**Results:**

We show that LD2-LD4 expression leads to the displacement of FAK and PYK2 from focal adhesions, blocking both enzymatic and non-enzymatic activities. It also dramatically inhibits 2D cell migration, as well as invasion in vitro. Importantly, LD2-LD4 exerts promising anti-tumor effects and nearly abolishes the appearance of metastatic foci. Finally, we show that an LD monomer can also displace both FAK and PYK2 from FAs suggesting that organic molecules with high affinity for the FAT HPs could mimic the LD2-LD4 activity.

**Conclusions:**

Targeting the FAT domain hydrophobic patches of FAK/PYK2 is a highly effective inhibitory strategy that can overcome the limitations of existing ATP competitive inhibitors and lead to the development of novel inhibitors with strong antitumor and antimetastatic activity.

**Supplementary Information:**

The online version contains supplementary material available at 10.1186/s12964-025-02203-1.

## Background

The Focal Adhesion Kinase (FAK) is a highly conserved non-receptor tyrosine kinase involved in several cellular processes primarily as a transducer of integrin and growth factor signaling, including regulation of cell adhesion, migration, growth, survival and gene expression [1, 2]. FAK is recruited to focal adhesions (FAs) via interaction of its FAT domain with LD-motif containing proteins, such as Paxillin [3, 4]. Activation of FAK begins with interaction of inactive FAK dimers with PI(4,5)P2-rich membranes, leading to disruption of the auto-inhibition conferred by the interaction between the FERM and kinase domains and exposure of Tyrosine 397 (Y397) [5]. Upon Y397 phosphorylation, FAK acts as a scaffold to recruit Src kinases at FAs, leading to phosphorylation of FAK on Y576 and Y577 in the activation loop, as well as recruitment and phosphorylation of downstream targets, such as the p130 Cas adaptor protein [6, 7]. Therefore, FAK exerts both kinase-dependent and kinase-independent (scaffolding) functions [4, 7]. In addition to its critical roles at FAs, FAK can also be found in the nucleus, where it functions by scaffolding transcriptional regulators, such as p53, and regulating expression of genes that are significant determinants of both the tumor micro-environment and anti-tumor immunity [8, 9].

Even though FAK is rarely mutated in cancer, it is very commonly overexpressed in various human malignancies [10–12], with > 20% of cancers including lung, breast, ovarian and colorectal, having an increased copy number of FAK [12–14]. This has been associated with poor overall patient survival [10, 11]. Therefore, and given the very low levels of expression in normal tissues [15], FAK has emerged as a promising therapeutic target for cancer and metastasis, with several small molecule FAK inhibitors undergoing preclinical and clinical trials [2].

PYK2 is a closely related paralogue of FAK, with which shares not only a high amino acid (48%) and structural similarity (65%) [16], but also several binding partners, endogenous activation and control mechanisms, and nuclear localization and export signals [17, 18]. However, PYK2 is weakly targeted to FAs, despite the interaction of its FAT domain with the LD motifs of Paxillin [12, 19]. Therefore, PYK2 and FAK are often overexpressed and regulate overlapping signaling pathways in cancer progression, such as the Wnt/β-catenin pathway [20]. Upon FAK inhibition, PYK2 can compensate for the loss of FAK, and its expression and phosphorylation are increased [8, 21, 22].

To date, all FAK or dual FAK/PYK2 inhibitors tested in clinical trials are ATP-competitive inhibitors that target the kinase domain and block the enzymatic activity by competing for ATP-binding [2]. These small molecule inhibitors exhibited limited clinical efficacy, without exerting significant clinical response and thus, recent work is now focusing on evaluating FAK kinase inhibitors in combination with other targeted therapies to overcome failure of chemotherapeutic agents and enhance the efficacy of immune-based treatments [12, 23, 24]. Scaffolding functions of FAK have also been implicated in cancer cell survival and metastasis, however exclusive targeting of FAK scaffolding functions does not appear to be sufficient as an anti-cancer or anti-metastatic treatment [25, 26]. Therefore, it is becoming clear that targeting both the kinase-dependent and kinase-independent functions of FAK could significantly improve clinical efficacy outcomes. The potential benefit of targeting both enzymatic and non-enzymatic functions of FAK is highlighted by work using proteolysis targeting chimeras (PROTACs) [27] which have shown promising results, yet they are still hampered by notable issues, including poor solubility and permeability, as well as poor cell penetration and oral bioavailability due to their large molecular weight [28, 29]. Therefore, this raises the necessity for the development of novel small molecule dual FAK/PYK2 inhibitors that can target both scaffolding and enzymatic functions of both proteins at the same time.

We have previously developed a novel strategy for the inhibition of FAK, that can effectively and simultaneously target both its kinase-dependent and kinase-independent functions. The strategy relies on the displacement of FAK from FAs, using a peptide, to compete with interactions taking place between the LD motifs of Paxillin and the hydrophobic pockets (HPs) of the FAK FAT domain, shown to be necessary and sufficient for FAK´s FA targeting [17, 30]. Specifically, we showed that a short peptide containing the LD2 and LD4 motifs of Paxillin (LD2-LD4), as well as an LD2 or LD4 dimer, are sufficient to mask interactions at the HPs of the FAT domain of FAK, leading to the displacement of the endogenous protein from FAs [31]. Herein, we further show that the LD2-LD4 containing peptide targets both FAK and PYK2 in different cancer cell types. We further examined the efficacy of this newly developed strategy for the inhibition of FAK and PYK2, by exploring the impact of LD2-LD4 expression on tumor growth and metastasis in vivo, using a highly metastatic breast cancer xenograft model. Collectively, data from our preclinical experiments indicated that expression of the LD2-LD4 peptide inhibited tumor growth. More importantly, however, LD2-LD4 expression had a dramatic impact on tumor progression and metastasis, nearly abolishing the appearance of metastatic tumors, highlighting the potential of this developed strategy as a therapeutic approach against cancer and metastasis.

## Methods

### Experimental design

We investigated the impact of LD2-LD4 expression on PYK2’s FA localization and activity utilizing FAK null, U-118 MG and MDA231-LM2-4175 stable cells inducibly expressing LD2-LD4, in co-immunoprecipitation experiments, Western Blot analysis and quantitative immunofluorescence, as well as 2D migration and 3D invasion assays to examined LD2-LD4 impact on tumor cell migration and invasion in vitro. We performed the preclinical experiments of this study utilizing a highly metastatic breast cancer xenograft model, followed by immunohistochemical analysis, for exploring LD2-LD4 impact on tumor establishment, growth and metastasis.

### Plasmids

mScarletI-PYK2 construct was generated in two steps as followed: firstly, the sequence of mScarletI was amplified with PCR using EMTB-mScarletI (Addgene, #137801) as a template with the primers F:aaaGGATCCatggtgagcaagggcgaggcagtgat and R:aaaGAATTCcttgtacagctcgtccatgccgccgg, without a stop codon. The PCR program was as follows: 5 min at 95 °C for initial denaturation, followed by 35 cycles of 15 s at 95 °C, 30 s at 65 °C, 45 s at 68 °C and final extension at 68 °C for 5 min. The PCR product was cloned into an empty pCS108 vector with BamHI and EcoRI. Next, the sequence of human full-length wild type PYK2 was amplified with PCR with the primers F:aaaGAATTCatgtctggggtgtccgagcccctgagtcgag and R:aaaTCTAGActactctgcaggtgggtgggccagattggcc using the pEGFP-C3-FLAG-PYK2 WT plasmid as a template, kindly provided by Dr. Francisco Sánchez-Madrid Laboratory (Spain) [32]. The PCR program was as follows: 5 min at 95 °C for initial denaturation, followed by 35 cycles of 15 s at 95 °C, 30 s at 65 °C, 3:08 min at 68 °C and final extension at 68 °C for 5 min. The PCR product was cloned into the pCS108-mScarletI (no stop) vector with EcoRI and XbaI, in frame with mScarletI. mRuby-PYK2 plasmid was generated similarly to mScarletI-PYK2 plasmid by cloning of PYK2 insert into a pCS108-mRuby vector with same restriction enzymes.

The generation of GFP-LD2-LD4, GFP-LD2 and GFP-LD4 was previously described [31].

pCS2+ +GFP-Paxillin plasmid was used as a template for the generation of GFP-LD2-LD3 (139-233aa), GFP-LD3 (213–232aa), GFP-LD3-LD4 (214-279aa) and Linker(3–4)-LD4 (234-279aa) constructs. PCR amplification was performed with the following primers:

- F/LD2_NotI
- aaaGCGGCCGCGGTGGTGGTGGTGGTGGTggcagcaac
- F/LD3_NotI
- aaaGCGGCCGCGGTGGTGGTGGTGGTGGTgtgcgtcccagtgtggaga
- R/LD3_XhoI_STOP
- aaaCTCGAGCTAgactgggcttggcacagagctctccagctcatccag
- R/LD4_XhoI_STOP
- gggCTCGAGTTAgaacttaaagtcagagagggacgccatcagc
- F/LINKER34_NotI
- aaaGCGGCCGCGGTGGTGGTGGTGGTGGTcctgcaatcactgtgagccaaggggaggtga

PCR fragments were added to pcs108-GFP vector with NotI and XhoI.

### Cell lines, cell culture and transient transfection

FAK null, MDA-MB-231 (MDA), HeLa and HEK293T cell lines were obtained from ATCC, U-118 MG cell line was kindly provided by Dr. Pavlos Costeas Laboratory (CSHM, Karaiskakio Foundation) and MDA231-LM2-4175 (LM2), SUM149 and SUM159 cell lines were kindly provided by Dr. Panagiotis Papageorgis Laboratory (European University of Cyprus). SUM149 and SUM159 cells were cultured at 37 °C with 5% CO_2_ in DMEM/F12 (Gibco, #11320033) medium supplemented with 5% Fetal Bovine Serum (PAN BIOTECH, #P30-3306), 5 µg/mL insulin (Capricorn Scientific, #INS-K), 1 μg/mL hydrocortisone (Sigma-Aldrich, #H0135) and 1% 1XAntibiotic-Antimycotic (Biosera, #XC-A4110/100). All other cell lines were cultured at 37 °C with 5% CO_2_ in Dulbecco’s Modified Eagle’s Medium (Santa Cruz Biotechnology, #sc-224478) supplemented with 10% Fetal Bovine Serum and 1XAntibiotic-Antimycotic. Transient transfection with Lipofectamine 2000 (Invitrogen, #11668019) was performed in Opti-MEM reduced serum media (Gibco, #31985070) for 6 h, according to the manufacturer’s protocol. Cells were seeded in 15 mm round (Marienfeld, #0111550) HCl-treated glass coverslips 24 h before transfection. All cell lines were routinely tested for mycoplasma.

### GFP and LD2-LD4 stable cell line generation

Lentivirus-based stable cell line generation was performed as previously described [31]. Single-cell cloning isolation was performed for MDA-LD2-LD4 and LM2-LD2-LD4 cells to derive cells with uniform high expression of LD2-LD4.

### Growth curves

Approximately 200 000 cells were seeded onto triplicate 12-well plates (Santa Cruz Biotechnology, #sc-204444) in DMEM supplemented with 10% FBS and cell growth curves were generated by harvesting cells every day by trypsinization, followed by manually cell counting with Neubauer Chamber (Reichert Bright-Line Metallized Hemacytometer, Hausser Scientific), as previously described [33]. Imaging was performed with a Zeiss Axiovert 200M microscope with a Plan-APOCHROMAT 10x/0.45 Ph1 objective and a Zeiss AxioCam MRm camera.

### Cell immunostaining and imaging

Cells seeded on HCl-treated glass coverslips were rinsed with 1XPBS (Gibco, #10010–015) and fixed with 4% PFA (Sigma-Aldrich, #158127-5G) as previously described [31]. Alternatively, cells were fixed and permeabilized with 100% ice-cold methanol (Sigma-Aldrich, #M1775-1GA) for 20 min at − 20 °C or with 1:1 mixture of 4% PFA and 0.5% Triton X-100 (Sigma-Aldrich, #1610407) in 1XPBS for 1 min on ice, followed by 5-min fixation in 4% PFA. All fixations were followed by three 5-min washes with IXPBS and subsequent blocking with 10% donkey serum (Jackson Immumoresearch, #017–000–121) in 1XPBS for 30 min and incubated with primary antibodies overnight at 4 °C in blocking solution. Primary antibodies used in immunofluorescence experiments were: goat anti-GFP (1:1000; Novus, #NB100-1770), rabbit anti-PYK2 (1:100; Invitrogen, #700183), rabbit anti-phospho-Y402_PYK2 (1:500; Invitrogen, #44-618G), mouse anti-Vinculin (1:500; Santa Cruz Biotechnology, #sc-73614), rabbit anti-Vinculin (1:2000; Proteintech, #26520-I-AP), mouse anti-FAK (1:1000; Proteintech, #66258-1-Ig), rat anti-FAK (1:500; BioLegend, #694002), mouse anti-Paxillin (1:500; R&D Systems, #AF4259), rabbit anti-FAK (1:1000; Proteintech, #12636-1-AP), rabbit anti-ILK (1:500; Abcam, #ab74336), goat anti-Talin C- 20 (1:500; Santa Cruz, #sc-7534), rabbit anti-phosho-Y397_FAK (1:500; Invitrogen, #44-624G), rabbit anti-phosho-Y576_FAK (1:500; Invitrogen, # 44652G), rabbit anti-phosho-Y31_Paxillin (1:500; Santa Cruz, #sc-14035), rabbit anti-phosho-Y118_Paxillin (1:500; Invitrogen, #44-722G) and mouse anti-p-Tyr (pY20) (1:500; Santa Cruz, #sc-508). Primary antibodies were washed off with three 5-min washes in 1XPBS, followed by subsequent incubation with secondary antibodies in blocking solution for 1.5 h at room temperature. Secondary antibodies used were: DyLight™ 405 AffiniPure Donkey Anti-Mouse IgG (H + L) (1:100; Jackson ImmunoResearch, #715–475-150), Donkey anti-Goat IgG (H + L) Cross-Adsorbed Secondary Antibody, Alexa Fluor™ 488 (1:500; Invitrogen, #A-11055), Donkey anti-Mouse IgG (H + L) Highly Cross-Adsorbed Secondary Antibody, Alexa Fluor™ 568 (1:250; Invitrogen, #A10037), Donkey anti-Rabbit IgG (H + L) Highly Cross-Adsorbed Secondary Antibody, Alexa Fluor™ 568 (1:250; Invitrogen, #A10042), Donkey anti-Rat IgG (H + L) Highly Cross-Adsorbed Secondary Antibody, Alexa Fluor™ 568 (1:250; Invitrogen, #A78946), Donkey anti-Mouse IgG (H + L) Highly Cross-Adsorbed Secondary Antibody, Alexa Fluor™ 647 (1:100; Invitrogen, #A-31571), Donkey anti-Rabbit IgG (H + L) Highly Cross-Adsorbed Secondary Antibody, Alexa Fluor™ 647 (1:100; Invitrogen, #A-31573), Donkey anti-Goat IgG (H + L) Cross-Adsorbed Secondary Antibody, Alexa Fluor™ 647 (1:100; Invitrogen, #A-21447) and Donkey anti-Rat IgG (H + L) Highly Cross-Adsorbed Secondary Antibody, Alexa Fluor™ Plus 647 (1:100; Invitrogen, #A48272). Secondary antibodies were washed off with 1XPBS 3 times and coverslips were mounted inverted in ProLong™ Diamond Antifade Mountant (Thermo Scientific, #P36961). Confocal and super-resolution imaging was performed as previously described [31], using lasers 405nm, 488 nm, 561 nm and 647 nm.

### Cell lysis and preparation

#### For SDS-PAGE

Cells seeded on 6-well tissue culture plate (Santa Cruz Biotechnology, #sc-204443) were washed once with ice-cold 1XPBS and lysed with 2XSDS buffer [200 mM Tris pH 6.8, 20% glycerol (Thermo Scientific, #15514011), 4% SDS (Sigma, #436143 - 100G, 2% 2-mercaptoethanol (Sigma-Aldrich, #M3148), 0.2% Bromophenol blue (Fisher Scientific, #50–488–539] on ice, scraped with a cell scraper (Santa Cruz Biotechnology, #sc-395250) and collected in 1.5 ml Eppendorf tube (Santa Cruz Biotechnology, #sc-200271). Lysates were then passed through 1 ml 25G syringe (Pic solution, #02071250300350) multiple times, followed by incubation at 95^ο^C for 5 min and centrifugation (16 100 g for 10 min). Cleared lysates stored at − 20^ο^C.

#### For immunoprecipitation

Cells seeded on a 10 cm tissue culture plate (Santa Cruz Biotechnology, #sc-200286) were washed once with ice-cold 1XPBS and lysed with ice-cold RIPA buffer [20 mM Tris pH 7.5, 150 mM NaCl, 1% NP40 (Sigma, #74385)] supplemented with protease inhibitors (Roche, #11836153001), sodium orthovanadate (1 mM) (Sigma-Alrich, #S6508 - 10G) and PMSF Protease Inhibitor (1 mM) (Fluka, #78830) on ice, scraped with a cell scraper and collected in 1.5 ml Eppendorf tube. Lysates were then passed through 1 ml insulin 25G syringe multiple times, followed by centrifugation (16 100 g for 10 min). Cleared lysates were stored at − 20^ο^C.

### Immunoprecipitation

10μL of GFP-Trap Agarose bead slurry (Proteintech, #gta) were equilibrated 3 times with 500 μL ice-cold RIPA buffer, followed by centrifugation (2 500 g for 5 min) at 4 °C. Protein extracts of induced and uninduced cells were gently added to equilibrated beads and the mixture was rotated for 1.5 h at 4 °C. The beads were washed 5 times with ice-cold RIPA buffer and then resuspended in 10 μl 2XLaemli sample buffer (Bio-Rad, #1610737) supplemented with 2-mercaptoethanol (Sigma-Aldrich, #M3148). After a short spin, samples were boiled for 5 min at 95 °C and the immunocomplexes were isolated with centrifugation (2 500 g for 5 min) at 4 °C and analyzed by western blot.

### Focal adhesion isolation

Isolation of focal adhesion structures was performed according to Kuo et al., 2011 [34] by adding TEA-containing low ionic strength buffer in cultured LM2-LD2-LD4 stable cells (induced and uninduced) seeded on 10 cm cell culture dishes (2 × plates for each condition), followed by Western Blot analysis.

### Western blotting

Lysates were loaded into SDS–polyacrylamide gels, along with protein ladder marker (NIPPON Genetics, #MWP03) and then they were transferred onto nitrocellulose membrane (Porablot NCP- MACHERY-NAGEL, #741280). The membrane was blocked in 5% BSA (Sigma, #A9647-50G) in 1XPBS for 1 h, followed by overnight incubation with primary antibodies diluted in 5% BSA in 0.1% PBS-Tween 20 (1XPBS and 0.1% Tween 20 (Sigma, #P6585 - 100ML)) at 4 °C. Primary antibodies used for western blot analysis were: rabbit anti-GFP (1:3000; Proteintech, #50430–2-AP), mouse anti-GFP (1:500; Invitrogen, #A-11120), rabbit anti-phosho-Y31_Paxillin (1:1000; Santa Cruz, #sc-14035-R), rabbit anti-PYK2 (1:1000; Invitrogen, #700183), mouse anti-phospho-Y402_PYK2 (1:800; Santa Cruz, #sc-293142), mouse anti-PYK2 (1:1000; Santa Cruz, #sc-393181), rabbit anti-phospho-Y402_PYK2 (1:800; Invitrogen, #44-618G), mouse anti-α Tubulin (1:1000; Santa Cruz Biotechnology, #sc-5286), mouse anti-FAK (1:1000; Proteintech, #66258-1-Ig), rabbit anti-phosho-Y397_FAK (1:1000; Invitrogen #44-624G), rabbit anti-β-Actin (1:1000; Cell Signaling Technology, Inc., #8457), mouse anti-GAPDH (1:1000; Santa Cruz Biotechnology, #sc-32233) and mouse anti-p53 (1:1000; Santa Cruz Biotechnology, #sc-126). After three 10-min washes in 0.1% PBS-Tween 20, the membrane was incubated with secondary antibodies diluted in 5% BSA in 0.1% PBS-Tween 20 for 1 h at room temperature, followed by three 5-min washes in 0.1% PBS-Tween 20. HRP-conjugated secondary antibodies used were: a) for single visualization: m-IgGκ BP-HRP (1:5000; Santa Cruz Biotechnology, #sc-516102) and mouse anti-rabbit IgG-HRP (1:5000, Santa Cruz Biotechnology, #sc-2357), b) in combination with fluorescent antibodies: goat anti-rabbit IgG-HRP (1:5000; Santa Cruz Biotechnology, #sc-2054) and goat anti-mouse IgG-HRP (1:5000; Santa Cruz Biotechnology, #sc-2302). Fluorescent secondary antibodies used were: Donkey anti-Mouse IgG (H + L) Highly Cross-Adsorbed Secondary Antibody, Alexa Fluor™ Plus 800 (1:6000; Invitrogen, #A32789) and Donkey anti-Rabbit IgG (H + L) Highly Cross-Adsorbed Secondary Antibody, Alexa Fluor™ Plus 680 (1:6000; Invitrogen, #A32802). Immobilon Forte Western HRP substrate (Millipore, #WBLUF0500) was used as a chemiluminescent HRP detection reagent. Visualization was performed using the Bio-Rad ChemiDoc MP Imaging System.

### Live-cell imaging

#### Migration assay

Uninduced and pre-induced (48 h) LM2-LD2-LD4 cells or MDA-MB-231 cells transiently transfected with GFP-Linker(3-4)-LD4 construct were seeded in 2-well ibidi chamber slides (Ibidi, #80286) in 1XCO_2_ independent medium (Gibco, #18045088) supplemented with 10% FBS and 1XAntibiotic-Antimycotic. Live cell imaging was performed in triplicates for approximately 21.5 h utilizing ibidi heating system (Ibidi, #10927) on a Zeiss Axiovert 200 M microscope with a Plan-APOCHROMAT 10x/0.45 Ph1 objective and a Zeiss AxioCam MRm camera.

#### FA localization

Uninduced and pre-induced (48 h) LM2-LD2-LD4 cells transfected with mRuby-PYK2 were seeded in 2-well ibidi chamber slides in Leibovitz's L- 15 (Gibco, #11415064) medium supplemented with 10% FBS and 1XAntibiotic-Antimycotic. Live-cell imaging was performed as previously described [31], using Zeiss LSM 900 Airyscan 2 laser scanning confocal microscope (Carl Zeiss AG, Germany) with a PlanApochromat 63x/1.40 oil DIC immersion objective and lasers 488 nm and 561 nm.

#### Scratch-wound assay

Uninduced and induced (48 h) LM2-LD2-LD4 cells seeded and grown to confluence in 2-well ibidi chamber slides (Ibidi, #80286) and wounding was performed by scratching the monolayer of cells with a pipette tip. Live-cell imaging was performed in Leibovitz's L-15 (Gibco) medium supplemented with 1% FBS and 1XAntibiotic-Antimycotic for approximately 10 h on a Zeiss Axiovert 200 M microscope with a Plan-APOCHROMAT 10x/0.45 Ph1 objective and a Zeiss AxioCam MRm camera.

### Invasion assay

Invasion assay was performed as previously described [31] with few modifications. Uninduced LM2-LD2-LD4 cells were resuspended in a solution of 2% low serum collagen (Santa Cruz Biotechnology, #sc-136157) [107μL Collagen, 89μL DMEM supplemented with 1% FBS, 11μL 10XPBS (Gibco, #70011044), 5μL 1 M NaOH and 2μL 1XAntibiotic-Antimycotic] to achieve a final concentration of 66 000 cells/μL. 1.5 μL of the mixture was placed as a droplet at the center of a modified chamber gel invasion assay device developed in our lab [35]. A round 15 mm coverslip was added to the top and the collagen mixture was allowed to set in a humidifying chamber (37 °C) for 11.5 min. The remaining area under the round coverslip was filled with a second layer of a solution of 2% full serum collagen (107μL Collagen, 89μL DMEM supplemented with 10% FBS, 11μL 10XPBS, 5μL 1 M NaOH and 2μL 1XAntibiotic-Antimycotic), that was allowed to set in a humidifying chamber (37 °C) for 30 min. The device was then filled with DMEM supplemented with 10% FBS and 10 μg/ml doxycycline was added to the + DOX well. Static images were obtained with a Zeiss Axiovert 200 M microscope with an EC PLAN-APOCHROMAT 5x/0,16 objective and a Zeiss AxioCam MRm camera.

### Spheroids

Approximately 5000 cells were seeded in 96-well flat bottom plate (Corning, #3596) coated with 1% agarose, in full DMEM medium supplemented with 10% FBS and 1XAntibiotic-Antimycotic, followed by centrifugation 5 min x 1000 rpm. After 24 h-incubation, full cell medium was removed with 1 ml syringe and 2% full serum collagen (107μL Collagen, 89μL DMEM supplemented with 10% FBS, 11μL 10XPBS, 5μL 1 M NaOH and 2μL 1XAntibiotic-Antimycotic) (10 μg/ml doxycycline was added to the + DOX mixture) was added carefully to cells. Upon 30 min incubation in a humidifying chamber (37 °C), full DMEM medium was added to cells. Static images were obtained with a Zeiss Axiovert 200 M microscope and a Zeiss AxioCam MRm camera.

### Tumor xenografts

NOD/SCID female immunodeficient mice (8–12 weeks old) used for the in vivo experiments were provided by The Cyprus Institute of Neurology and Genetics.

#### MDA-GFP xenografts

A total of 1X10^6^ MDA-GFP cells were suspended in IXPBS and injected orthotopically into the left mammary fat pad of each NOD/SCID immunodeficient mouse (*n* = 10 mice), following anesthetization of mice with IP injection of Avertin (20 mg/kg). Mice received either diet standard food pellets (Mucedola s.r.l, #4RF25) (*n* = 5 mice) or 0.625 g/kg doxycycline food pellets (Safe Custom Diets, #E8200 Version 0115) (*n* = 5 mice). Throughout the experiment, tumor volume was measured using a caliper (Fine Science Tools, #30087–20) and calculated by the equation: x = 1/2*(length*width^2^). At the end of the study (30 days) mice were sacrificed via CO_2_ inhalation and excised tissues were fixed and processed for immunohistochemical analysis.

#### LM2-GFP xenografts

LM2-GFP xenografts were generated in 2 independent experiments similarly to MDA-GFP xenografts with few modifications. A total of 2X10^6^ LM2-GFP cells were injected into mice (*n* = 16) and half of mice received diet standard food and the other half 0.625 g/kg doxycycline food pellets. Mice were sacrificed five weeks post-injections.

#### LM2-LD2-LD4 xenografts

LM2-LD2-LD4 xenografts were generated in 3 independent experiments, similarly to MDA-GFP xenografts with few modifications. A total of 2X10^6^ LM2-LD2-LD4 cells were injected into mice that were either a) pre-induced to express LD2-LD4 using doxycycline ex vivo for 6 days prior to tumor cell injection (‘PRE-INDUCED’ group), or induced to express LD2-LD4 via doxycycline food pellets (0.625 g/kg) in vivo, starting either (b) on the day of tumor cell injection (‘DOX DAY 0’ group) (prophylactic treatment) or c) 7 days post tumor cell injection (‘DOX DAY 7’ group) (therapeutic treatment) or d) not induced to express LD2-LD4 at any point (‘-DOX’ control group) (*N* = 6–7 mice per group). Mice were sacrificed nine weeks post-injections.

### Immunohistochemistry

Tissues collected from mice were washed 3 times in 1XPBS and pre-fixed with 4% PFA overnight at 4 °C. Samples were then washed 3 times in 1XPBS for 5 min and embedded in PolyFreeze (Sigma-Aldrich, #P0091) in cryomolds (Thermo Fisher Scientific, #2219) and frozen completely at − 80 °C. 19 μm thick cryosections were produced using the SLEE MAINZ cryostat and SuperfrostTMPlus Adhesion Microscope slides (Epredia, #J1800AMNZ) were used for the mounting of sections. Freshly section tissues dried at room temperature for 5–6 h and stored at − 80 °C. All pre-fixed tissues were imaged prior to their cryopreservation with a Zeiss Lumar.V12 stereoscope and a Zeiss NeoLumar S 1.5 × FWD 30 mm lens, using a Zeiss AxioCam MRc5 camera. 

**Fluorescent immunohistochemistry** was performed by two 5-min washes in 1XPBS, fixation of the tissues with 4% PFA for 10 min, quenching with 10 mM Glycine (pH = 7.8) (Sigma-Aldrich, #G8898-500G) for 10 min and permeabilization with 0.2% Triton X- 100 in 1XPBS for 10 min at room temperature. Sections were then washed 2 times with 1XPBS and incubated in blocking solution (10% Donkey serum in 0.2% Triton in 1XPBS) for 1 h at room temperature and then immunostained with rat anti-integrin beta1 (CD29) antibody, clone mAb13 (1:500; Merck, #MABT821) overnight at 4 °C. Tissues were next washed 2 times with 1XPBS and incubated with Donkey anti-Rat IgG (H + L) Highly Cross-Adsorbed Secondary Antibody, Alexa Fluor™ 568 (1:250; Invitrogen, #A78946) for 1.5 h in blocking buffer. Next, sections were washed twice with 1XPBS for 5 min and incubated in Hoechst 33342 (1:5000; Invitrogen, #62249) in 1XPBS for 20 min at room temperature. Sections were mounted with ProLong™ Diamond Antifade Mountant (Thermo Fisher Scientific, #P36961) and covered with a glass rectangular coverslip 24X50 mm (Marienfeld, #0101222). Confocal imaging of immunostained tissues was performed on a Zeiss LSM 900 laser scanning confocal microscope with an EC Plan-Neofluar 10x/0.30 M27 or a PLAN APOCHROMAT 20x/0.8 objective using lasers 405 nm, 488 nm and 561 nm.

**Chromogenic Immunohistochemistry** was performed by two 5-min washes in 1XPBS, fixation of the tissues with 4% PFA for 20 min, quenching with 10 mM Glycine (pH = 7.8) for 20 min and permeabilization with 0.2% Triton X-100 in 1XPBS for 20 min at room temperature. Sections were then washed 2 times with 1XPBS and incubated in 3% Hydrogen Peroxide (EMSURE, #1.07210.1000) for 10 min, followed by two 5-min washes in 1XPBS. Next, tissues were incubated in blocking buffer [10% goat serum (Jackson Immumoresearch, #005–000–121)] in 1% BSA in 1XPBS for 10 min at room temperature and then immunostained with anti-mAb13 in 0.1% BSA in 1XPBS overnight at 4 °C. Sections were next washed 2 times with 1XPBS and incubated with Goat anti-Rat IgG-HRP (1:700; Santa Cruz, #sc-2006) for 10 min in 0.1% BSA in 1XPBS. Next, sections were washed twice with 1XPBS for 5 min and incubated in DAB Chromogen Solution (DAB Substrate Kit; Abcam, #ab64238) according to the manufacturer’s protocol. Lastly, sections were washed with 1XPBS twice and then mounted in Fluoromount™ Aqueous Mounting Medium (Sigma-Aldrich, #F4680 - 25ML). Imaging of the tissues was performed with a Zeiss Lumar.V12 stereoscope and a Zeiss NeoLumar S 1.5 × FWD 30 mm lens, using a Zeiss AxioCam MRc5 camera.

### Quantifications and statistical analysis

Quantification of Western blot bands for 3 independent experiments was performed with ImageJ 1.52p. Quantification for the FA-localization of individual proteins was performed with Imaris 9.1.2. image analysis software. Specifically, a manual selection of each FA was performed in individual cells, and for quantification purposes, the mean cytosolic fluorescent intensity was subtracted from the mean FA fluorescent intensity for each protein. Live-cell time-lapse analysis of 2D migration assay (3 independent experiments) was performed with automated tracking of the motion path of each cell over time using Imaris software. Specifically, quantification of the mean track displacement length (μm) and mean track speed (μm/sec) of individual cells was automatically calculated by the software. Axiovision LE (AxioVs40 V 4.8.2.0) software was used for the quantification of the invasion efficiency of induced and uninduced LM2-LD2-LD4 cells, using obtained static pictures. Specifically, the distance between the boundary of the inner and outer gel and the point that representative leading invading cells from different areas of the setup had reached was measured to compare the distance covered by induced and uninduced cells. Quantification of lung metastasis was performed with AxioVision LE software, utilizing images obtained following chromogenic immunohistochemistry application. Specifically, imaging analysis was performed by calculating the mean metastatic area/total lung area ratio in 13 sections per group from 2 independent in vivo experiments. Statistical analysis and graphs were performed with the GraphPad Prism software (Prism 5 For Windows, Version 5.02). All graph data are shown as mean values, while error bars represent S.E.M. Statistical analysis was performed using either two-tailed unpaired t-test, one-way ANOVA and Tukey's Multiple Comparison Test or regular two-way ANOVA test (Bonferroni post-test), with 95% confidence interval.

## Results

### LD2-LD4 directly interacts and effectively displaces both FAK and PYK2 from FAs

**Fig. 1:**
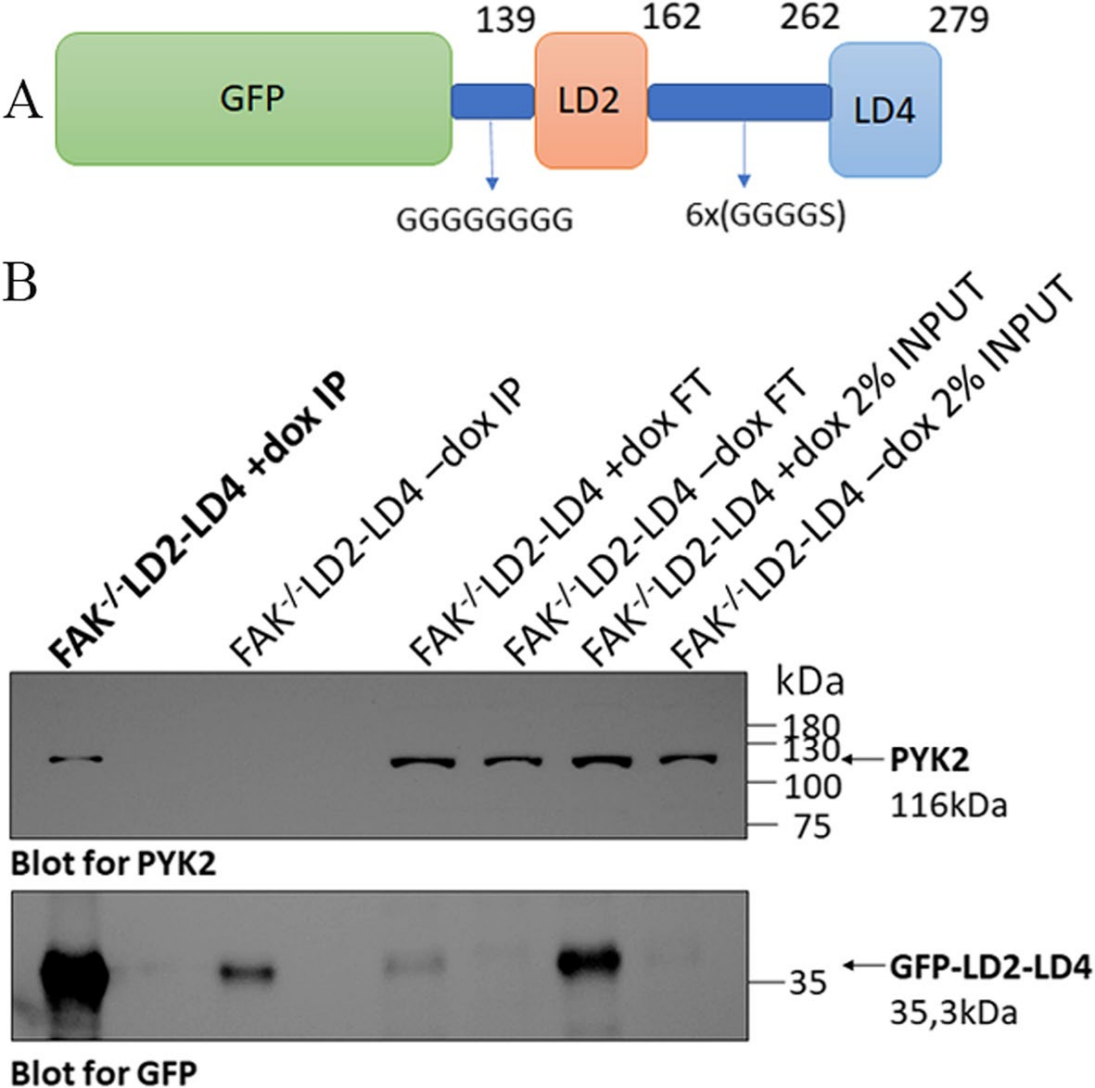
PYK2 immunoprecipitation from FAK null cells either inducibly expressing GFP-LD2-LD4 or not (control). **A** Schematic illustration of the LD2-LD4 peptide structure. **B** Western blot analysis following co-immunoprecipitation assay, showing interaction between LD2-LD4 and endogenous PYK2 in FAK null cells. PYK2 was immunoprecipitated from FAK null cells either inducibly expressing GFP-LD2-LD4 or not (control). Membranes were blotted for PYK2 (upper panel) and GFP (lower panel). PYK2 co-precipitation was observed only in cells inducibly expressing GFP-LD2-LD4. FT: flowthrough, IP: Immunoprecipitation

We previously generated a peptide that consisted of the LD2 (aa139 - 162) and LD4 (aa262 - 279) motifs of Paxillin linked via a synthetic, flexible linker composed of 6X GGGGS motifs (30aa) and fused to Green Fluorescent Protein (GFP) via a short stretch of 8 Glycines (GGGGGGGG) [31] (Fig. 1A). Expression of LD2-LD4 prevented FAK localization at FAs by competing with endogenous Paxillin for FAK binding [31]. PYK2 is also involved in cell migration, cancer development and metastasis and can compensate for the loss of FAK [21, 22]. PYK2, like FAK, interacts with Paxillin through the binding of its FAT domain to the LD2 and LD4 motifs [18], raising the possibility that LD2-LD4 expression could also target PYK2, establishing this approach as the sole dual-function, dual-target strategy, targeting both enzymatic and scaffolding functions of both proteins at the same time. We thus used lentiviral transduction to generate stable cell lines expressing GFP fused LD2-LD4, under the control of a tetracycline-dependent promoter [36], which allows inducible expression upon addition of doxycycline. We first generated stable FAK null (FAK^−/−^) fibroblasts inducibly expressing either GFP fused LD2-LD4 (hereunto referred to as ‘FAK^−/−^-LD2-LD4’) or GFP only (hereunto referred to as ‘FAK^−/−^-GFP’) (Additional file 1: Fig. S1A). FAK null fibroblasts are ideal for the investigation of the impact of LD2-LD4 expression directly on endogenous PYK2’s FA localization, expression and downstream targeting, given PYK2’s elevated expression in these cells [37], as well as the absence of possible secondary effects emanating from the displacement of FAK from FAs. In addition, use of FAK null fibroblasts eliminates complications stemming from the cross reactivity of antibodies, especially phospho-specific ones, against FAK and PYK2.

**Fig. 2:**
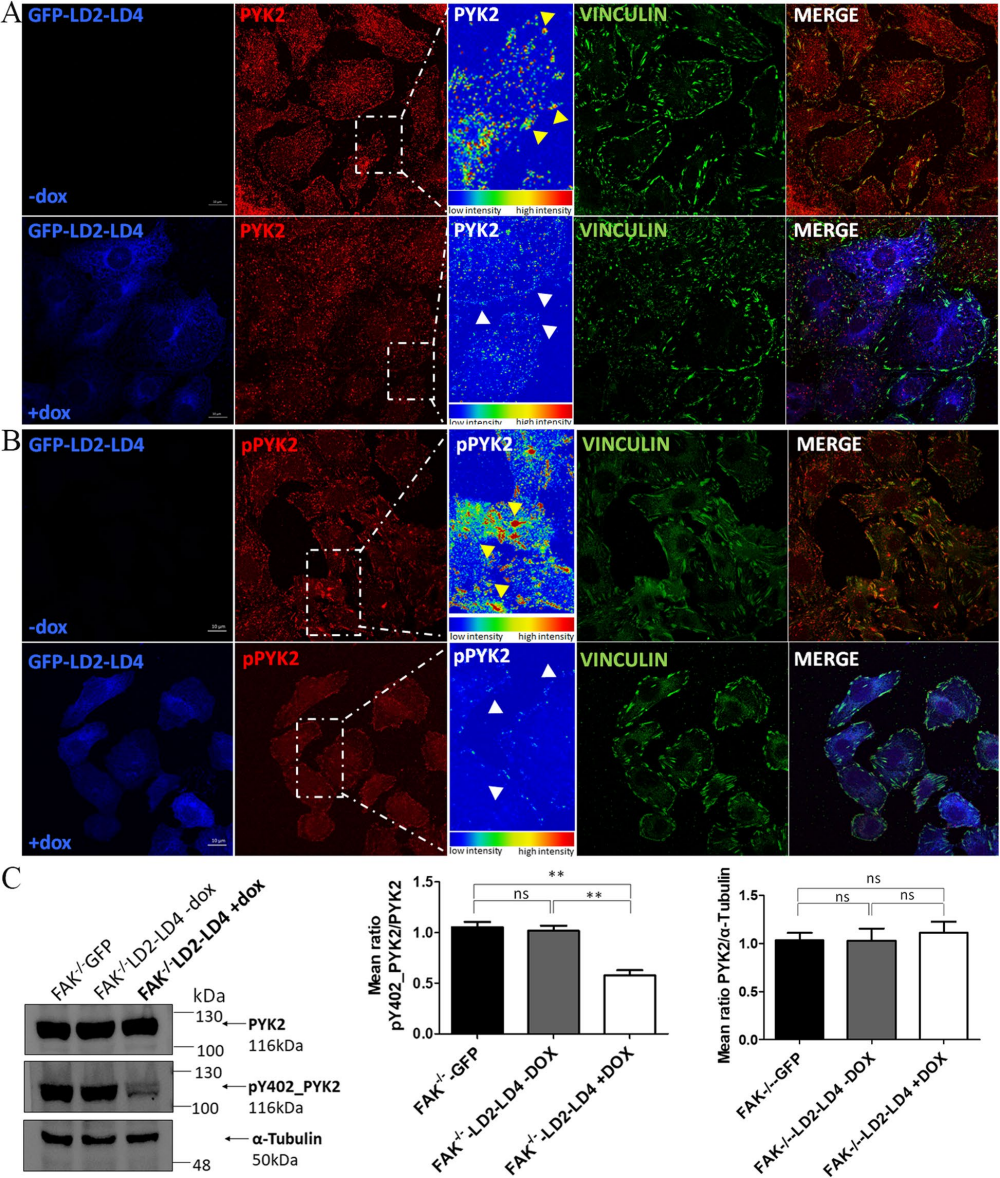
LD2-LD4 effectively displaces PYK2 from FAs and reduces phosphorylation of Y402. **A**, **B** Representative super-resolution images of FAK null cells (GFP-LD2-LD4 expressors and non-expressors) immunostained for GFP, PYK2 and Vinculin (**A**) or pY402_PYK2 and Vinculin (**B**). Yellow arrows point to FAs of GFP negative cells, and white arrows point to FAs of GFP positive cells. Expression of PYK2 and pY402_PYK2 is presented as intensity color-coded images, in zoom-in panels. LD2-LD4 effectively displaces PYK2 from FAs and reduces Y402 phosphorylation of PYK2 at FAs in expressors. Scale bars: 10 μm. **C** Western Blot and quantification indicating the phosphorylation status of PYK2 Y402, in FAK null cells, either inducibly expressing LD2-LD4 or not (control) and GFP control cells. Quantification of the mean ratio of phosphorylated PYK2 over total PYK2, using two-tailed unpaired t test, shows reduction of Y402 phosphorylation, upon LD2-LD4 induction. The mean ratio is 1.053 ± 0.05239 for GFP expressing cells, 1.017 ± 0.05207 for uninduced LD2-LD4 cells and 0.5767 ± 0.05207 for induced LD2-LD4 expressing cells (*N* = 3 independent experiments). LD2-LD4 expression does not affect total PYK2 expression levels. The mean ratio is 1.033 ± 0.07688 for GFP expressing cells, 1.030 ± 0.1253 for uninduced LD2-LD4 cells and 1.110 ± 0.1168 for induced LD2-LD4 expressing cells (*N* = 3 independent experiments). SEM is represented by error bars. **: *P* < 0.01, ns: not significant

We initially examined whether LD2-LD4 would directly interact with PYK2, in co-immunoprecipitation experiments performed using FAK null fibroblasts. As depicted in Fig. 1B, the LD2-LD4 peptide interacts with PYK2 as expected, raising the possibility that expression of LD2-LD4 could potentially displace PYK2 from FAs. Using indirect immunofluorescence and staining against PYK2 and Vinculin, as an FA marker previously shown to be unaffected by the expression of LD2-LD4 [31], we here show that induction of LD2-LD4 effectively displaces PYK2 from these complexes in FAK null fibroblasts (*P* < 0.0001) (Fig. 2A, Additional file 1: Fig. S1B). Given the impact of LD2-LD4 on PYK2 localization, we then wanted to determine whether LD2-LD4 expression could potentially inhibit kinase-dependent functions of PYK2 as effectively as it inhibited FAK kinase activity. Tyrosine 402 (Y402) in PYK2 is analogous to Y397 in FAK and thus serves as the primary autophosphorylation site [38]. Specifically, it provides a docking site for the SH2 domain of c-Src and leads to phosphorylation of PYK2 at Y579 and Y580 in the kinase domain activation loop [38, 39]. As demonstrated, upon LD2-LD4 expression, phosphorylation of Y402 is dramatically reduced in LD2-LD4 expressing cells compared to adjacent control cells (*P* < 0.0001) (Fig. 2B, Additional file 1: Fig. S1C). Given the inability to ensure that the phospho-specific PYK2 antibody only detected phosphorylated tyrosines on PYK2, we repeated the above-described experimental set-up using induced and uninduced FAK null cells, followed by Western Blot application, and confirmed a significant drop of PYK2 phosphorylation upon LD2-LD4 expression (GFP compared to + DOX: *P* = 0.0030; -DOX compared to + DOX: *P* = 0.0039) (Fig. 2C). The above data therefore show that LD2-LD4 expression leads to effective displacement of PYK2 from FAs and inhibition of its kinase activity.

Next, we investigated the impact of LD2-LD4 expression on PYK2’s FA localization and activation in cells that express high levels of both FAK and PYK2. While both FAK and PYK2 are ubiquitously expressed, PYK2 usually displays low expression in most cell types, with the exception of hematopoietic and neuronal tissues [18, 40]. Since FAK also displays elevated expression in neuronal cells [41], we generated stable U-118 MG human glioblastoma cells that inducibly express GFP fused LD2-LD4 (hereunto referred to as ‘U-118 MG-LD2-LD4’) to examine the effects of LD2-LD4 expression on both endogenous FAK and PYK2 (Additional file 1: Fig. S2A).

**Fig. 3:**
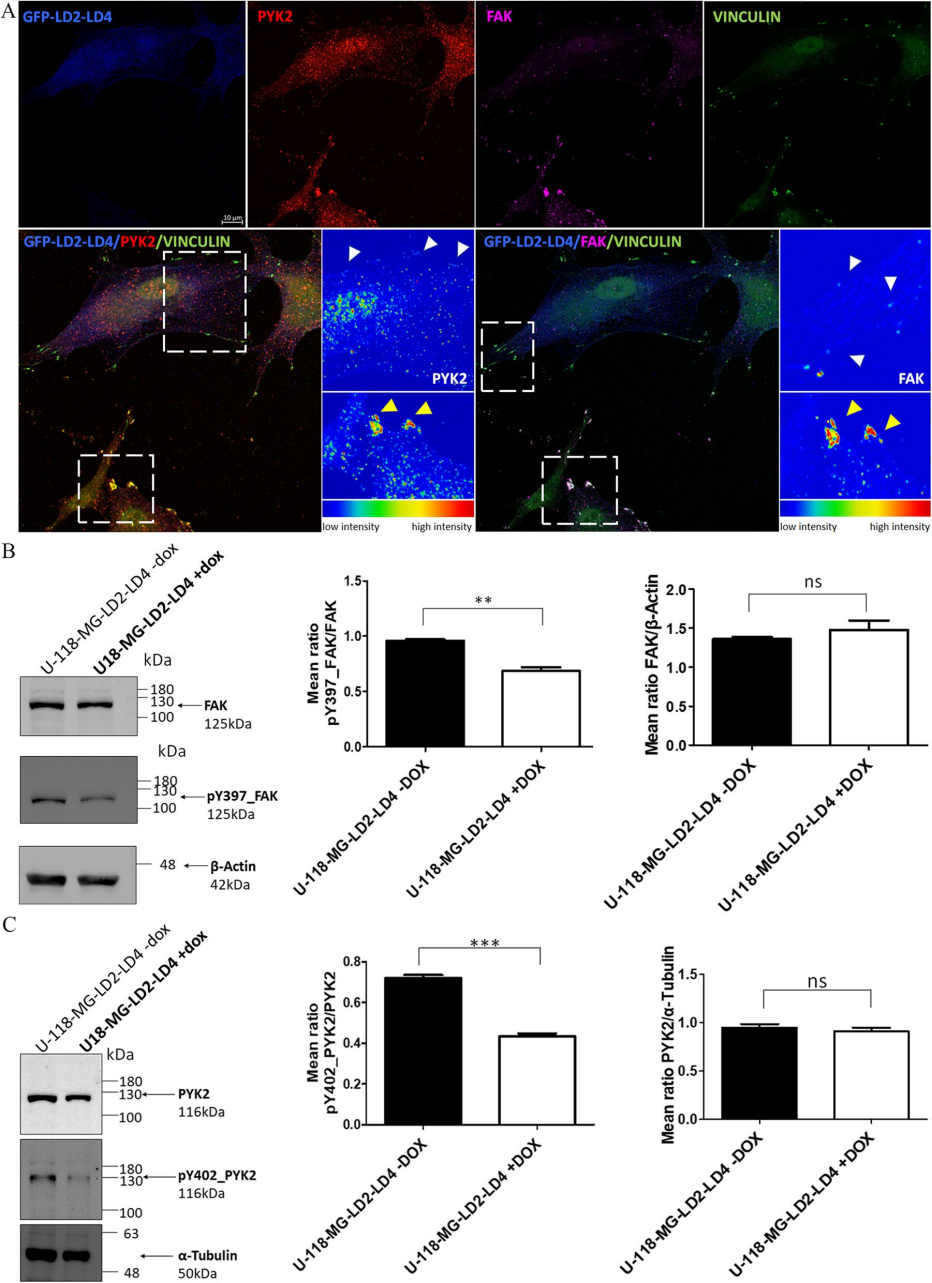
LD2-LD4 efficiently targets and inhibits both FAK and PYK2, simultaneously. **A** Representative super-resolution images of a mixed-cell population (GFP-LD2-LD4 expressors and non-expressors) of U-118 MG cells immunostained for GFP, PYK2, FAK and Vinculin. Expression of PYK2 and FAK are presented as intensity color-coded images, in zoom-in panels. Yellow arrows point to FAs of GFP negative, and white arrows point to FAs of GFP positive cells. Expression of LD2-LD4 effectively displaces both endogenously expressed FAK and PYK2 from FAs. Scale bars: 10 μm. **B**, **C** Western Blot and quantification indicating the phosphorylation status of FAK Y397 (**B**) and PYK2 Y402 (**C**), in stable U-118 MG cells either inducibly expressing LD2-LD4 or not (control). Quantification of the ratio of (**B**) phosphorylated FAK over total FAK (mean ratio is 0.9600 ± 0.01155 for uninduced LD2-LD4 and 0.6900 ± 0.03055 for induced LD2-LD4 expressing cells), as well as (**C**) phosphorylated PYK2 over total PYK2 (mean ratio is 0.7200 ± 0.01528 for uninduced LD2-LD4 and 0.4333 ± 0.01453 for induced LD2-LD4 expressing cells) (*N* = 3 independent experiments). LD2-LD4 induction results in reduced phosphorylation of Y397 and Y402 sites on FAK and PYK2, respectively. LD2-LD4 expression does not affect total PYK2’s or FAK’s total expression levels. **B** The mean ratio is 1.360 ± 0.02656 for uninduced LD2-LD4 cells and 1.477 ± 0.1217 for induced LD2-LD4 expressing cells (*N* = 3 independent experiments). **C** The mean ratio is 0.9500 ± 0.03606 for uninduced LD2-LD4 cells and 0.9100 ± 0.03786 for induced LD2-LD4 expressing cells (*N* = 3 independent experiments). SEM is represented by error bars. **: *P* < 0.01, ***: *P* < 0.001

As shown in Fig. 3A, using anti-FAK and anti-PYK2 specific antibodies [rat anti-FAK (BioLegend, #694002) and rabbit anti-PYK2 (Invitrogen, #700183)] (Additional file 1: Fig. S2B), both endogenous FAK and PYK2 are effectively displaced from FAs in U-118 MG-LD2-LD4 expressing cells (FAK, PYK2: *P* < 0.0001) (and Additional file 1: Fig. S2C, S2D), upon LD2-LD4 expression. Moreover, expression of LD2-LD4 also lead to a significant reduction in the phosphorylation of Y397 (Fig. 3B) and Y402 (Fig. 3C), the major autophosphorylation sites of FAK and PYK2 respectively, indicating that it effectively blocks the kinase activity of both proteins simultaneously (FAK: *P* < 0.01; PYK2: *P* < 0.001). Collectively, our data show that LD2-LD4 efficiently targets and inhibits both kinases at the same time.

### LD2-LD4 inhibits both FAK and PYK2 and reduces the proliferation of MDA231-LM2-4175 breast adenocarcinoma cells

**Fig. 4:**
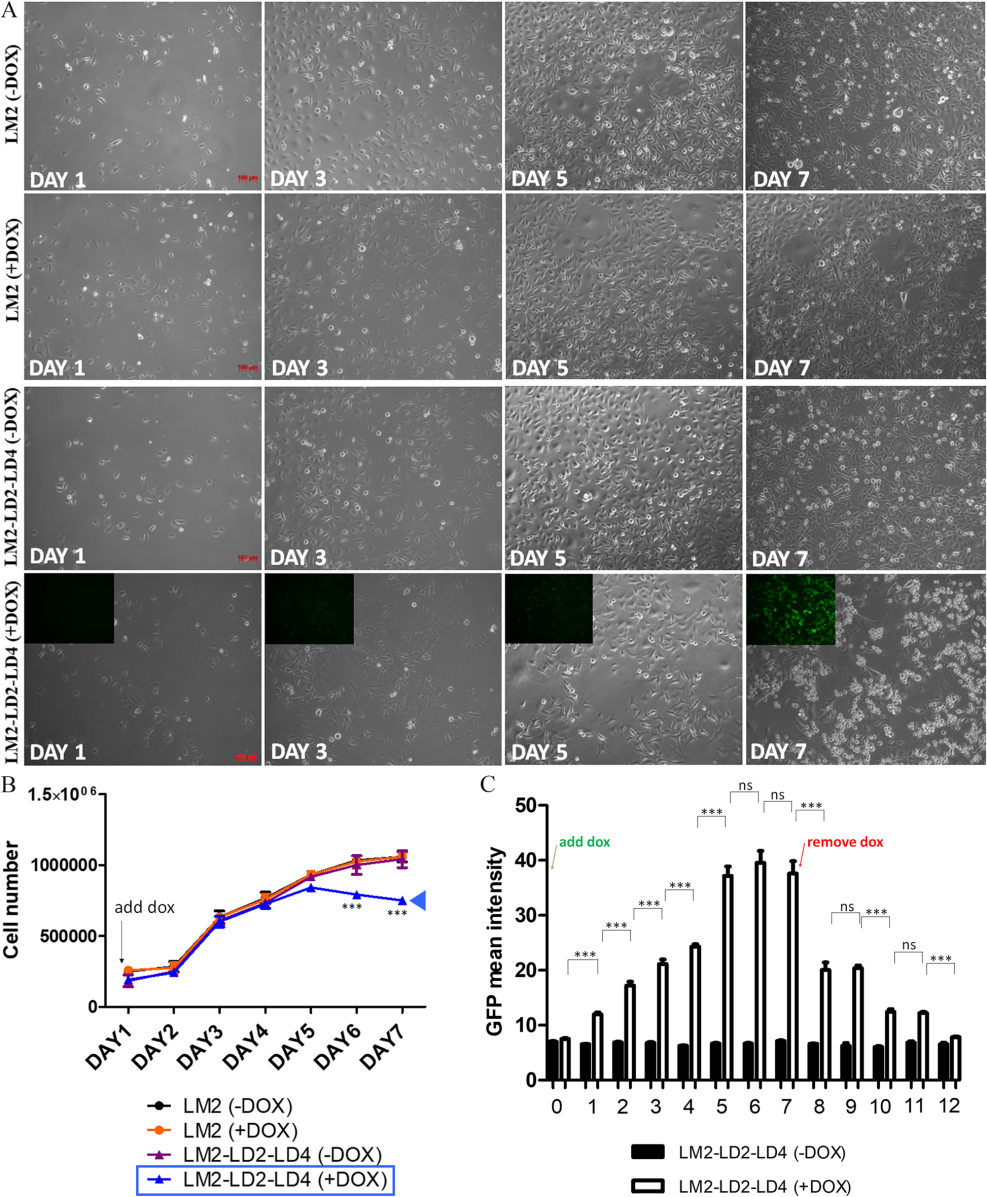
LD2-LD4 expression suppresses the proliferation of LM2 cells. **A**, **B** Representative microscope fields and growth curves of MDA231-LM2-4175 (LM2) cells and stable LM2 cells inducibly expressing LD2-LD4. Smaller images in 4^th^ panel are showing GFP expression of induced LM2-LD2-LD4 cells. LM2 cells are not doxycycline sensitive. LD2-LD4 expression significantly inhibits LM2 cell growth and viability, 6 days post doxycycline induction. Scale bars: 100 μm. Regular two-way ANOVA test (Bonferroni post-test) was used for multiple group comparison. SEM is represented by error bars. ***: *P* < 0.001. **C** GFP mean intensity graph showing LM2-LD2-LD4 GFP expression levels upon addition and removal of doxycycline. Two-tailed unpaired t test was used for the quantification of GFP levels of induced LM2-LD2-LD4 cells between two consecutive days (DAY0 compared to DAY1: P*** < 0.0001, DAY1 compared to DAY2: P*** < 0.0001, DAY2 compared to DAY3: P*** 0.0004, DAY3 compared to DAY4: P*** 0.0005, DAY4 compared to DAY5: P*** < 0.0001, DAY5 compared to DAY6: P 0.3898, DAY6 compared to DAY7: P 0.5457, DAY7 compared to DAY8: *P* *** < 0.0001, DAY8 compared to DAY9: P 0.7945, DAY9 compared to DAY10: *P* *** < 0.0001, DAY10 compared to DAY11: P 0.6846, DAY11 compared to DAY12: *P* *** < 0.0001. *N* = total 2255 cells. SEM is represented by error bars. ns: not significant

FAK has been shown to exert a critical role in breast cancer initiation, progression and metastasis [42]. PYK2 is also aberrantly expressed in breast cancer and several studies have shown that, similar to FAK, aberrant expression of PYK2 promotes breast cancer cell proliferation, migration, invasion, metastasis, and chemo-resistance [43]. We have previously shown that LD2-LD4 expression efficiently blocked FAK’s enzymatic and scaffolding functions, leading to inhibition of cell migration and tumor cell invasion in vitro, using the MDA-MB-231 breast adenocarcinoma cell line [31]. In this work, we further show that LD2-LD4 expression was effective in simultaneously inhibiting the kinase activity of both FAK and PYK2. Thereby, we wanted to examine the potential of this approach in vivo, in a breast cancer preclinical mouse model. However, and in agreement with previous reports [44], both our in vitro and in vivo experiments using MDA-MB-231 cells expressing GFP-fused LD2-LD4, under a doxycycline inducible promoter, and control GFP expressing cells (hereunto referred to as ‘MDA-LD2-LD4’ and ‘MDA-GFP’, respectively), indicated that doxycycline alone had a strong antiproliferative effect on these cells making them a poor choice for exploring this strategy in vivo (Additional file 1: Fig. S3A-S3C). Specifically, no significant difference was observed between the proliferation rate of MDA-GFP + dox control cells and MDA-LD2-LD4 + dox cells in vitro (*P* = 0.3798) (Additional file 1: Fig. S3B. Additionally, mice injected with MDA-GFP cells and treated with doxycycline were unable to grow tumors larger than 0.5 mm^3^, in comparison with non-treated mice which developed tumors with maximum volume 7.8 mm^3^ in 31 days (Additional file 1: Fig. S3C). We thus turned to a derivative cell line, MDA231-LM2-4175 cells (hereunto referred to as ‘LM2’), established from the parental MDA-MB-231 cell line after 3 passages in nude mice and shown to have a higher metastatic potential than the parental line, especially for forming lung metastasis [45]. Importantly, as shown in Fig. 4, LM2 cells do not display any sensitivity to doxycycline in vitro (Fig. 4A, B) or in vivo (Additional file 1: Fig. S4B-S4E, S5A-S5B), unlike the parental line. LM2 cells were thus selected for all subsequent experiments including xenografts. We generated LM2 cells expressing either GFP alone (as a control) (hereunto referred to as ‘LM2-GFP’) or GFP fused to LD2-LD4 under a doxycycline inducible promoter and selected specific clones with high GFP expression, in order to derive cells that uniformly express high levels of LD2-LD4 (hereunto referred to as ‘LM2-LD2-LD4’) (Additional File 1: Fig. S4A). We subsequently examined the impact of LD2-LD4 expression on LM2 proliferation in vitro*.* As shown LD2-LD4 expression led to a significant reduction of LM2 cell proliferation (*P* < 0.001) when peak LD2-LD4 expression levels were reached (DAY 6) and dramatic change in cell morphology the day after with a large percentage of cells rounding up (Fig. 4B, C).

**Fig. 5:**
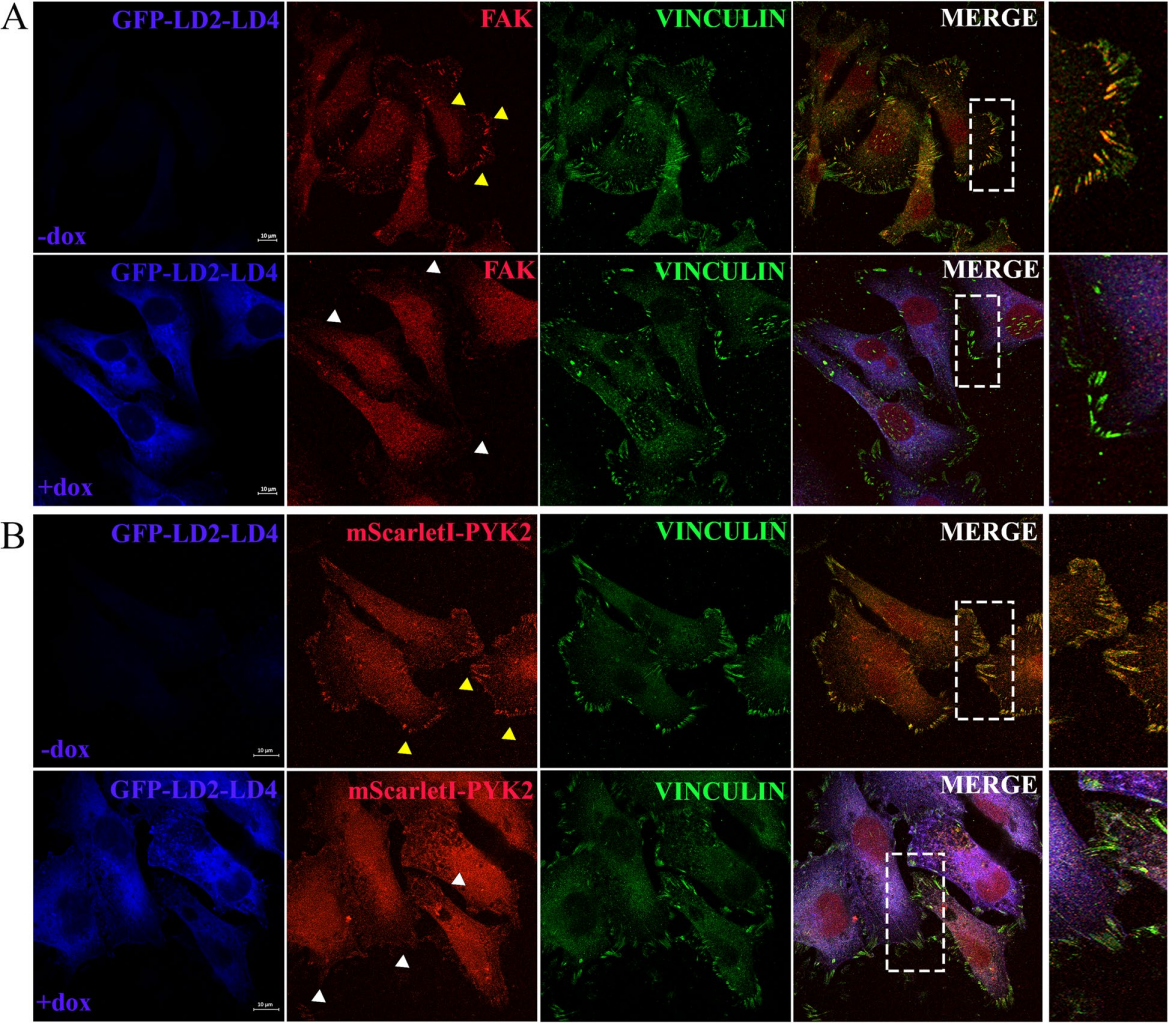
LD2-LD4 effectively displaces both FAK and PYK2 from FAs, in LM2 cells. **A**, **B** Representative confocal images of LM2 cells inducibly expressing LD2-LD4, immunostained for FAK (**A**) and Vinculin (**A**, **B**). **B** LM2-LD2-LD4 cells were transiently transfected with mScarletI-PYK2 construct prior to LD2-LD4 induction. Yellow arrows point to FAs of control cells and white arrows point to FAs of LD2-LD4 expressing cells. Dashed white rectangles indicate zoomed-in areas, shown in the panels of the last (right-most) column. LD2-LD4 effectively displaces both FAK and PYK2 from FAs. Scale bars: 10 μm

We then proceeded to examine how localization of FAK and PYK2 in LM2 cells was affected by doxycycline-inducible expression of LD2-LD4, using immunofluorescence. As shown in Fig. 5A, doxycycline-induced expression of LD2-LD4 in LM2 cells lead to effective displacement of FAK from FAs. Given the low levels of PYK2 at FAs of these cells, we transiently expressed mScarletI-PYK2 in LM2 cells, to help better visualize the impact of LD2-LD4 expression on PYK2 localization. As shown in Fig. 5B, transiently expressed PYK2 is effectively displaced from FAs, upon LD2-LD4 induction. We further evaluated the impact of LD2-LD4 on PYK2’s localization at FAs, using live-cell imaging. Specifically, we transiently expressed mRuby-PYK2 in LM2-LD2-LD4 cells and we verified PYK2’s displacement in doxycycline-induced cells (Additional file 1: Fig. S6A). Additionally, we confirmed the impact of LD2-LD4 expression on PYK2 levels at FAs biochemically. As shown in Additional file 1: Fig. S6B, LD2-LD4 leads to a significant reduction of PYK2 association with FAs.

Importantly, LD2-LD4 expression did not affect FA localization of other core proteins, including Vinculin (Fig. 5A, B), Paxillin (Additional file 1: Fig. S7A), ILK (Additional File 1: Fig. S7B), and Talin (Additional file 1: Fig. S7C), establishing the specificity of FAK/PYK2 targeting. In addition, LD2-LD4 expression reduced the phosphorylation levels of FAK tyrosine 397 (Additional file 1: Fig. S8A), but also the phosphorylation levels of downstream targets of FAK, such as Paxillin (Additional file 1: Fig. S8B, S8C) and total tyrosine phosphorylation at FAs (Additional file 1: Fig. S8D). Collectively, the data shows that LD2-LD4 expression leads to dual inhibition of both FAK and PYK2 in LM2-LD2-LD4 cells.

**Fig. 6:**
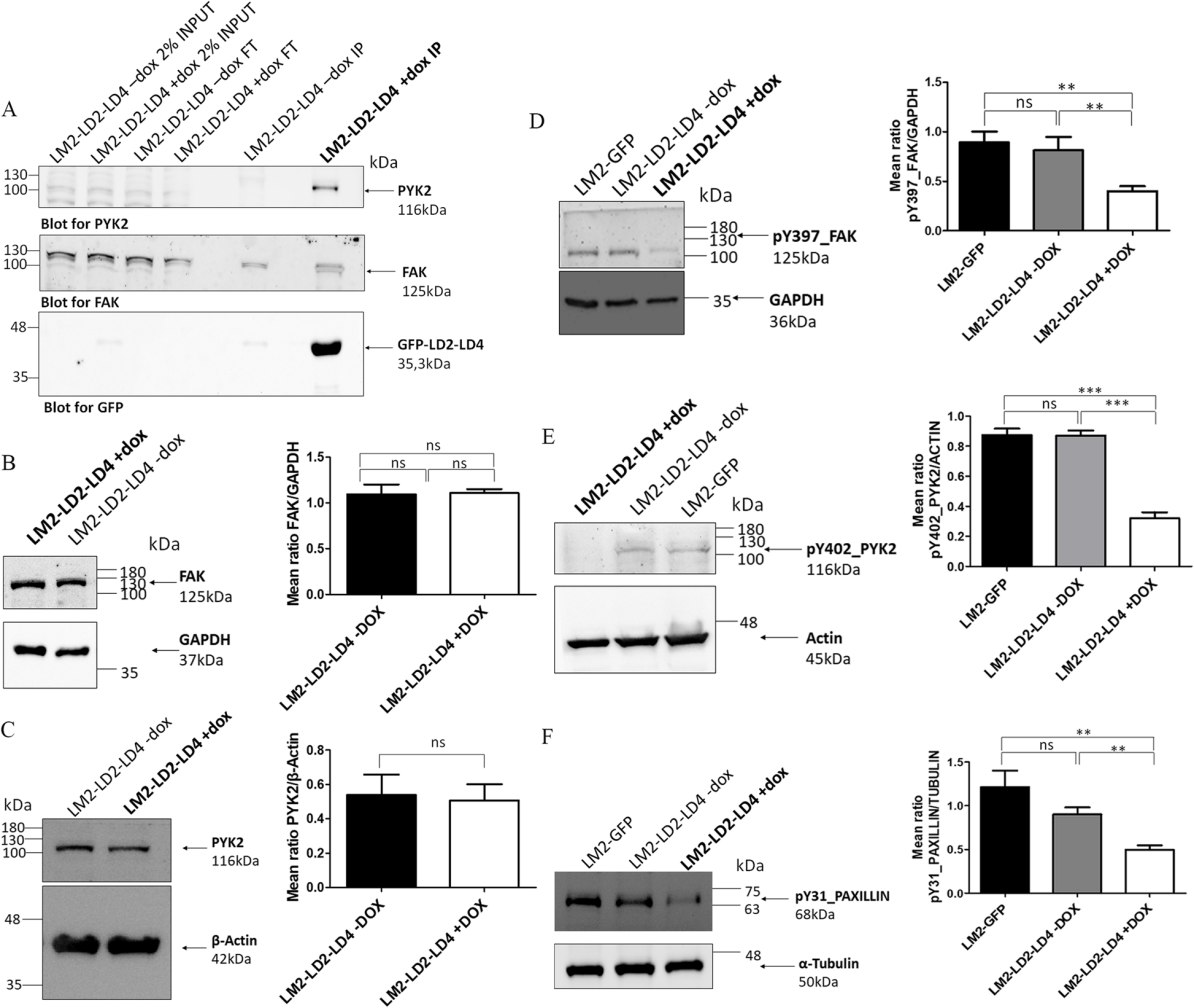
LD2-LD4 is efficiently targeting and inhibiting both FAK and PYK2 in LM2 cells. **A** Western Blot following an immunoprecipitation assay, showing LM2 cells either inducibly expressing GFP-LD2-LD4 or not (control), precipitated with anti-GFP and blotted for PYK2 (upper lane), FAK (middle lane) and GFP (lower lane). PYK2 and FAK are shown to directly interact with LD2-LD4. FT: Flowthrough, IP: Immunoprecipitation. **B**-**F** Representative Western Blot membranes and quantification, using LM2 cells stably expressing GFP or inducibly expressing LD2-LD4, show that LD2-LD4 expression does not affect (**B**) FAK or (**C**) PYK2 expression levels, but leads to a reduction in FAK phosphorylation on Y397 (**D**), PYK2 phosphorylation on Y402 (**E**) and Paxillin phosphorylation on Y31 (**F**). Graphs: Two-tailed unpaired t test was used for the quantification of (**B**) total FAK levels over GAPDH (mean ratio is 1.093 ± 0.1073 for uninduced and 1.107 ± 0.04410 for LD2-LD4 expressing cells), (**C**) total PYK2 levels over β-actin (mean ratio is 0.5381 ± 0.1197 for uninduced and 0.5062 ± 0.09544 for LD2-LD4 expressing cells) (**D**) total FAK Y397 levels over GAPDH (mean ratio is 0.9767 ± 0.07126 for GFP, 0.9500 ± 0.06506 for uninduced and 0.4000 ± 0.0500 for LD2-LD4 expressing cells) (**E**) total PYK2 Y402 levels over actin (mean ratio is 0.8733 ± 0.04333 for GFP, 0.8700 ± 0.03464 for uninduced and 0.3200 ± 0.04041 for LD2-LD4 expressing cells) and (**F**) total Paxillin Y31 levels over α-tubulin (mean ratio is 1.133 ± 0.1286 for GFP, 0.9233 ± 0.05364 for uninduced and 0.4733 ± 0.003333 for LD2-LD4 expressing cells), *N* = 3 independent experiments. SEM is represented by error bars. **: *P* < 0.01, ***: *P* < 0.001, ns: not significant

Given this result, and as reported in Fig. 6A, we used co-IP experiments and further validated that the observed inhibition was due to direct interaction of the LD2-LD4 peptide with the FAK and PYK2 kinases. Additionally, biochemical analysis confirmed that the induction of LD2-LD4 in LM2-LD2-LD4 cells did not affect FAK (*P* = 0.9140) (Fig. 6B) or PYK2 (*P* = 0.8452) (Fig. 6C) expression, but lead to a significant reduction of the phosphorylation of FAK (Y397) (GFP compared to + DOX: *P* = 0.0026; -DOX compared to + DOX: *P* = 0.0027) (Fig. 6D), PYK2 (Y402) (GFP compared to + DOX: *P* = 0.0007; -DOX compared to + DOX: *P* = 0.0005) (Fig. 6E), and Paxillin (Y31) (GFP compared to + DOX: *P* = 0.0068; -DOX compared to + DOX: *P* = 0.0011) (Fig. 6F). Therefore, the data show that this is a dual inhibitor approach, that effectively blocks activation of both FAK and PYK2, as well as their downstream signaling from FAs. Given that FAK binds to p53 in the nucleus and leads to a reduction of p53 levels [9], we also investigated the impact of LD2-LD4 expression on p53. Our results show that there is no significant change of p53 protein levels upon LD2-LD4 induction in LM2-LD2-LD4 cells (*P* = 0.6574) (Additional file 1: Fig. S9A).

### LD2-LD4 expression inhibits tumor cell migration and invasion in vitro

**Fig. 7:**
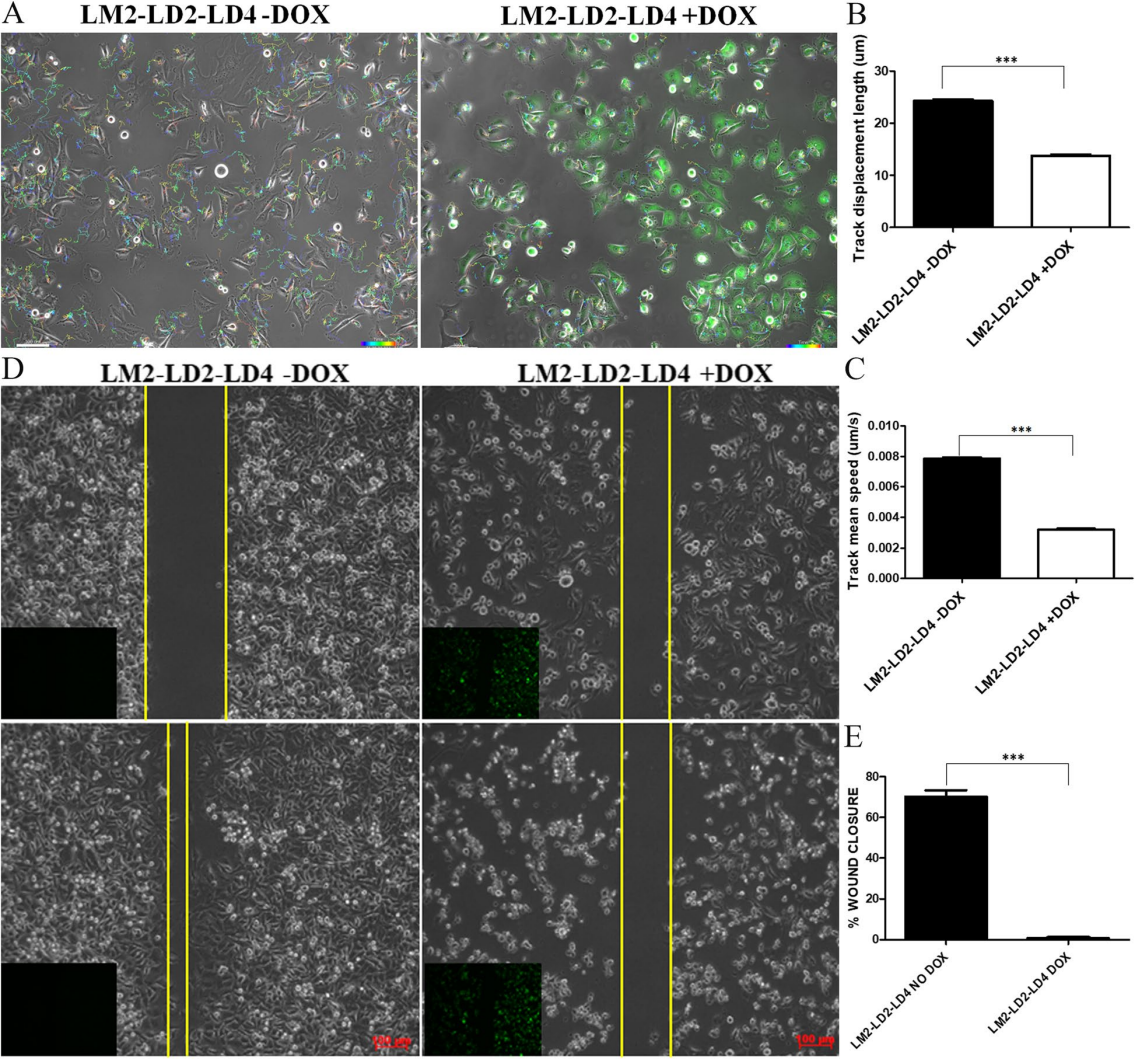
LD2-LD4 expression inhibits tumor cell migration in vitro. **A** Live-cell time-lapse analysis, using automated tracking of the motion path of each cell over time, indicates that expression of LD2-LD4 dramatically inhibits cell migration of LM2 cells. Scale bars: 100 μm. **B**, **C** Quantification of mean track displacement length (um) (**B**) and mean track speed (um/sec) (**C**) of LM2 cells expressing LD2-LD4 or not (control), using two-tailed unpaired t test. The mean track displacement length of uninduced control cells is 24.28 ± 0.3385 and for induced cells is 13.79 ± 0.1833. The mean track speed of uninduced control cells is 0.007854 ± 7.135e- 005 and for induced cells is 0.003176 ± 7.496e- 005. *N* = 3816 control, 3876 induced cells, *N* = 3 independent experiments. SEM is represented by error bars. ***: *P* < 0.0001. **D**, **E** Scratch-wound assay utilizing the LM2-LD2-LD4 cells (induced and uninduced) showing that LD2-LD4 expression is dramatically inhibiting cell migration. Smaller panels show GFP expression. Two-tailed unpaired t test was used for the quantification of the mean % wound closure (for uninduced control cells is 70.15 ± 3.292 and for induced cells is 0.5983 ± 0.1229). *N* = 3 independent experiments. SEM is represented by error bars. ***: *P* < 0.0001

The above data show that LD2-LD4 expression displaces both FAK and PYK2 from the FAs of LM2 cells, preventing their activation and subsequent phosphorylation of downstream targets. We therefore wanted to examine the functional implications of this approach related to 2D cell migration and 3D cell invasion. As shown in Fig. 7, LD2-LD4 expression significantly inhibits cell migration of LM2 cells, in addition to inhibiting cell growth and viability (Fig. 4A, B). Specifically, LD2-LD4 expressing cells manifested shorter track displacement lengths (*P* < 0.0001) (Fig. 7B) and moved at a reduced speed (*P* < 0.0001), compared to uninduced cells (Fig. 7C). In addition, we performed scratch-wound assay utilizing the LM2-LD2-LD4 cells (Fig. 7D, E), showing that LD2-LD4 expression is dramatically inhibiting cell migration and the ability of LM2 cells to close the wound (*P* < 0.0001). To ensure these results are not specific to the LM2 cell line, we performed 2D cell migration using two additional metastatic triple negative breast cancer cell lines, SUM149 and SUM159 [46]. As shown in Additional file 1: Fig. S9B-S9E, LD2-LD4 expression has a similar impact on both cell lines confirming that the peptide inhibitory activity on cell migration is not specific to LM2 cells.

**Fig. 8:**
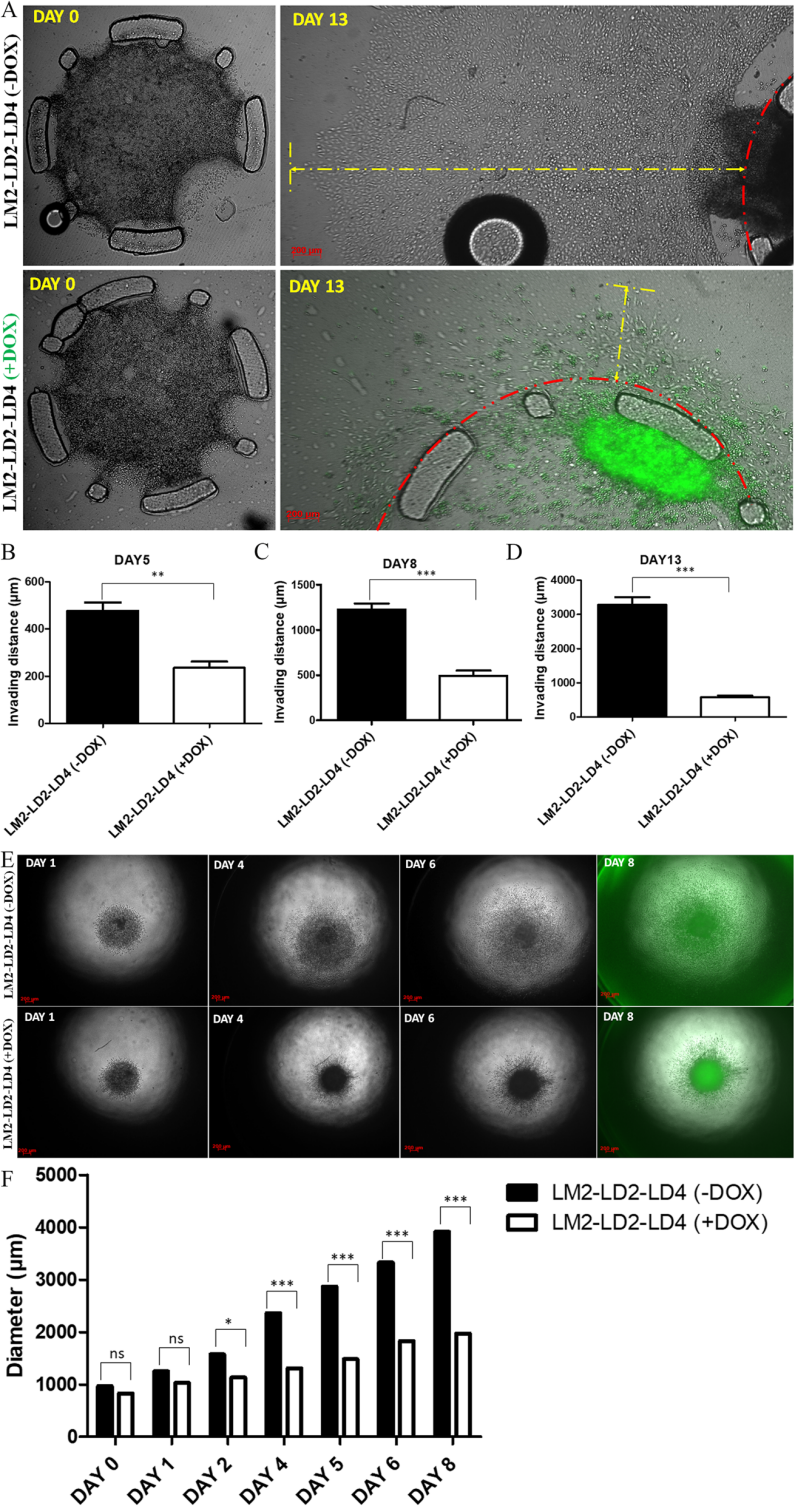
LD2-LD4 expression inhibits tumor cell invasion in vitro. **A** Imaging of LM2-LD2-LD4 cell invasion in a modified chamber gel invasion assay device at DAY0 and DAY13. LD2-LD4 inhibits tumor cell invasion. **B**-**D** Quantification of invading distance of LM2-LD2-LD4 cells [induced (+ dox) and control (-dox)], using static images obtained at days 5, 8 and 13 respectively, using two-tailed unpaired t test. **B** On day 5, the mean distance length of uninduced cells is 429.2 ± 41.40 and for induced cells is 235.2 ± 26.68. **C** On day 8, the mean distance length of uninduced cells is 1227 ± 66.11 and for induced cells is 494.3 ± 55.75. **D** On day 13, the mean distance length of uninduced cells is 3282 ± 223.5 and for induced cells is 574.9 ± 55.22. *N* = 3 independent experiments. SEM is represented by error bars. **: *P* value < 0.01, ***: *P* value < 0.0001. **E**, **F** Images obtained on DAY1, DAY4, DAY6 and DAY8 showing control LM2-LD2-LD4 spheroids displaying increased invasion, compared to induced LM2-LD2-LD4. Graph: Quantification of spheroid diameter, using regular two-way ANOVA test (Bonferroni post-test) for multiple group comparison. *N* = 6 spheroids per group. SEM is represented by error bars. *: *P* < 0.05, ***: *P* < 0.001. ns: not significant

We went on to address the impact of LD2-LD4 expression on tumor cell invasion utilizing a modified collagen invasion assay developed by our group, that accurately mimics the tumor microenvironment [35]. Invasion efficiency of induced LM2-LD2-LD4 cells compared to uninduced was quantified using static pictures, and as shown in Fig. 8A-D, LD2-LD4 induction led to approximately sixfold reduction of the invasive capacity of these cells (*P* < 0.0001). Additionally, we generated spheroids using the LM2-LD2-LD4 cells, verifying LD2-LD4 inhibitory effects on tumor cell invasion (Fig. 8E, F).

### LD2-LD4 expression has a dramatic impact on tumor progression and metastasis in vivo

**Fig. 9:**
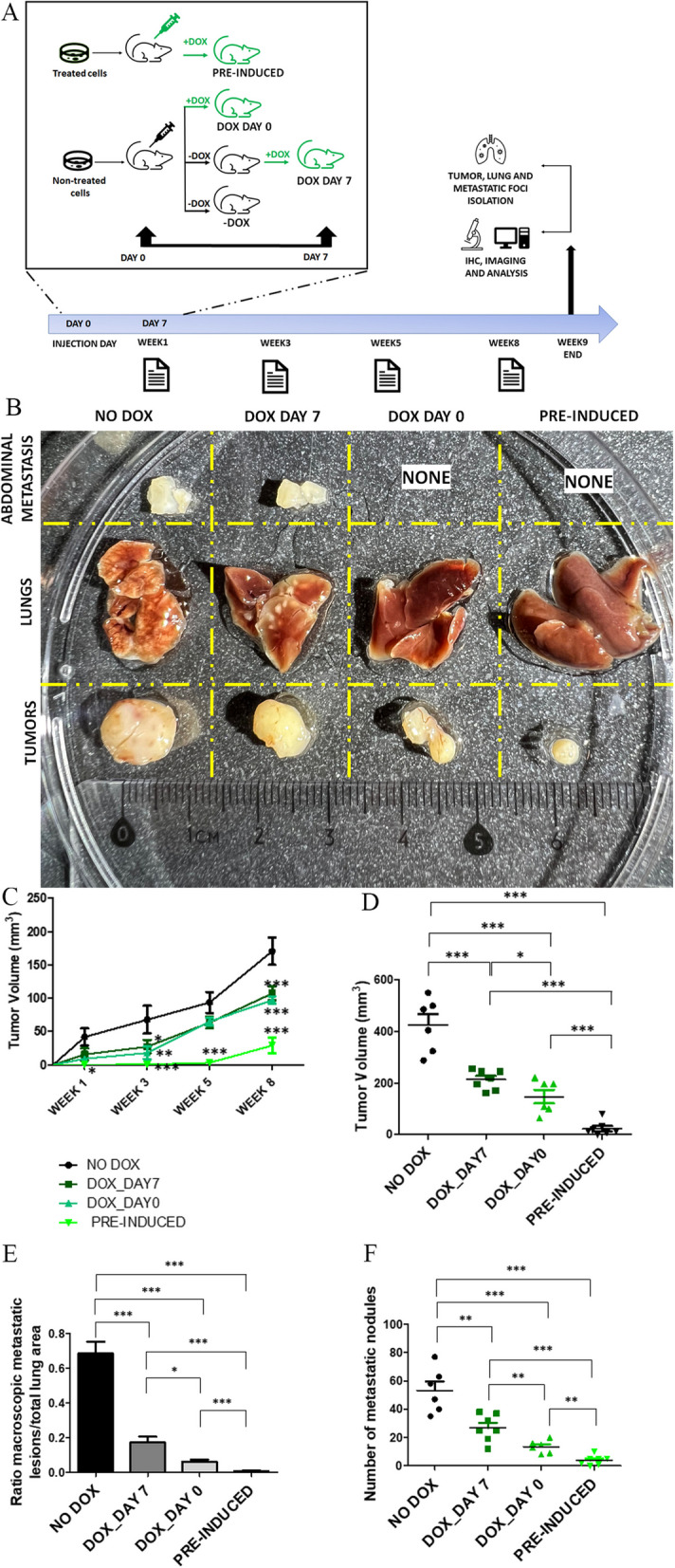
LD2-LD4 expression inhibits tumor growth and metastasis in vivo. **A** Schematic illustration of the experimental design and timeline used for the in vivo experiments. **B** Representative images showing tissues (tumors, lungs and abdominal metastatic foci) isolated 9 weeks post orthotopic injection of 2 × 10^6^ LM2 in the mammary fat pad of NOD/SCID mice. **C** Tumor growth curve showing inhibition of tumor growth upon LD2-LD4 induction. Regular two-way ANOVA test (Bonferroni post-test) was used for multiple group comparison. Black asterisks indicate significant difference between control (-DOX) and doxycycline-treated mice. SEM is represented by error bars. *N* = 6 mice in NO DOX group, 7 mice in DOX_DAY7 group, 6 mice in DOX_DAY0 group and 7 mice in PRE-INDUCED group. *: *P* < 0.05, **: *P* < 0.01, ***: *P* < 0.001. **D** Quantification of excised tumor volume of control compared to treated mice at 9 weeks, using two-tailed unpaired t test. The mean tumor volume of non-treated mice (NO DOX) is 425.5 ± 42.56, for the DOX_DAY7 group is 213.7 ± 14.28, for the DOX_DAY0 group is 147.4 ± 26.35 and for the PRE-INDUCED group is 23.29 ± 10.18. *N* = 6 mice in NO DOX group, 7 mice in DOX_DAY7 group, 6 mice in DOX_DAY0 group and 7 mice in PRE-INDUCED group. SEM is represented by error bars. *: *P* < 0.1, ***: *P* < 0.001. **E** Quantification of macroscopic lung metastasis, using stereoscope images and two-tailed unpaired t test. The mean ratio of metastatic area/total lung area of non-treated mice (NO DOX) is 0.6865 ± 0.06868 *N* = 6 mice, for the DOX_DAY7 group is 0.1726 ± 0.03469 *N* = 7 mice, for the DOX_DAY0 group is 0.06166 ± 0.01194 *N* = 6 mice and for the PRE-INDUCED group is 0.007814 ± 0.002802 *N* = 7 mice. SEM is represented by error bars. *: *P* = 0.0165, ***: *P* < 0.001. **F** Quantification of the number of lung metastatic nodules using two-tailed unpaired t test. The mean ratio of the number of lung metastatic nodules of non-treated mice (NO DOX) is 53.33 ± 6.401 *N* = 6 mice, for the DOX_DAY7 group is 26.86 ± 3.595 *N* = 7 mice, for the DOX_DAY0 group is 13.50 ± 1.839 *N* = 6 mice and for the PRE-INDUCED group is 4.000 ± 1.272 *N* = 7 mice. SEM is represented by error bars. **: P 0.0032 (NO DOX vs DOX_DAY7, P 0.0095 (DOX_DAY7 vs DOX_DAY0, P 0.0012 (DOX_DAY0 vs PRE-INDUCED, ***: P 0.0001 (NO DOX vs DOX_DAY0, *P* < 0.0001 (NO DOX vs PRE-INDUCED, DOX_DAY7 vs PRE-INDUCED)

At the molecular level, expression of LD2-LD4 led to displacement of both FAK and PYK2 from FAs. The functional implications of this displacement were the inhibition of LM2 cell proliferation, migration and invasion in vitro. We therefore wanted to investigate the anti-tumor and anti-metastatic potential of this approach in a preclinical setting, since this inhibitory strategy had never been tested in vivo. Given that we confirmed that MDA231-LM2-4175 cell tumor growth and metastatic potential are not affected by doxycycline, we used LM2 cells with doxycycline-inducible expression of LD2-LD4 in a xenograft mouse model and examined inhibition of tumor establishment, growth, and metastasis. We orthotopically injected immunocompromised NOD/SCID mice with 2 × 10^6^ LM2-LD2-LD4 cells in the left mammary fat pad that were either a) pre-induced to express LD2-LD4 using doxycycline ex vivo for 6 days prior to tumor cell injection (‘PRE-INDUCED’ group), or induced to express LD2-LD4 via doxycycline containing food pellets (0.625 g/kg) in vivo, starting either (b) on the day of tumor cell injection (‘DOX DAY 0’ group) (prophylactic treatment) or c) 7 days post tumor cell injection (‘DOX DAY 7’ group) (therapeutic treatment) or d) not induced to express LD2-LD4 at any point (‘-DOX’ control group) (Fig. 9A).

Collectively, data from three independent preclinical experiments showed that LD2-LD4 expression can inhibit tumor growth (Fig. 9B-D). As shown in Fig. 9C, the earlier the time of induction of LD2-LD4 expression the slower the rate of orthotopic tumor growth. Pre-induction of LD2-LD4 6 days prior to tumor cell injection resulted in tumors that were unable to grow even 5 weeks post-injection (sixfold reduction in tumor volume compared to control group, *P* < 0.001 and threefold compared to prophylactic and therapeutic treatment, *P* < 0.001). Prophylactic and therapeutic treatments were not as effective as pre-induction, yet they both equally delayed tumor growth significantly (1.6-fold reduction in tumor volume compared to control group; *P* < 0.001). Moreover, as shown in Fig. 9D, there was a clear difference in orthotopic tumor volume, with pre-induction resulting in markedly smaller tumors compared to all other treatments (18-fold reduction in tumor volume compared to control tumors, *P* < 0.0001; ninefold compared to therapeutic treatment, *P* < 0.0001; sixfold compared to prophylactic treatment, *P* = 0.0007). Prophylactic and therapeutic treatment also resulted in significantly smaller in size tumors compared to the control group (therapeutic treatment: twofold reduction in tumor volume compared to control, *P* = 0.0004; prophylactic treatment: threefold reduction compared to control, *P* = 0.0002). Overall, it was clear that LD2-LD4 expression had a significant impact on orthotopic tumor growth.

**Fig. 10:**
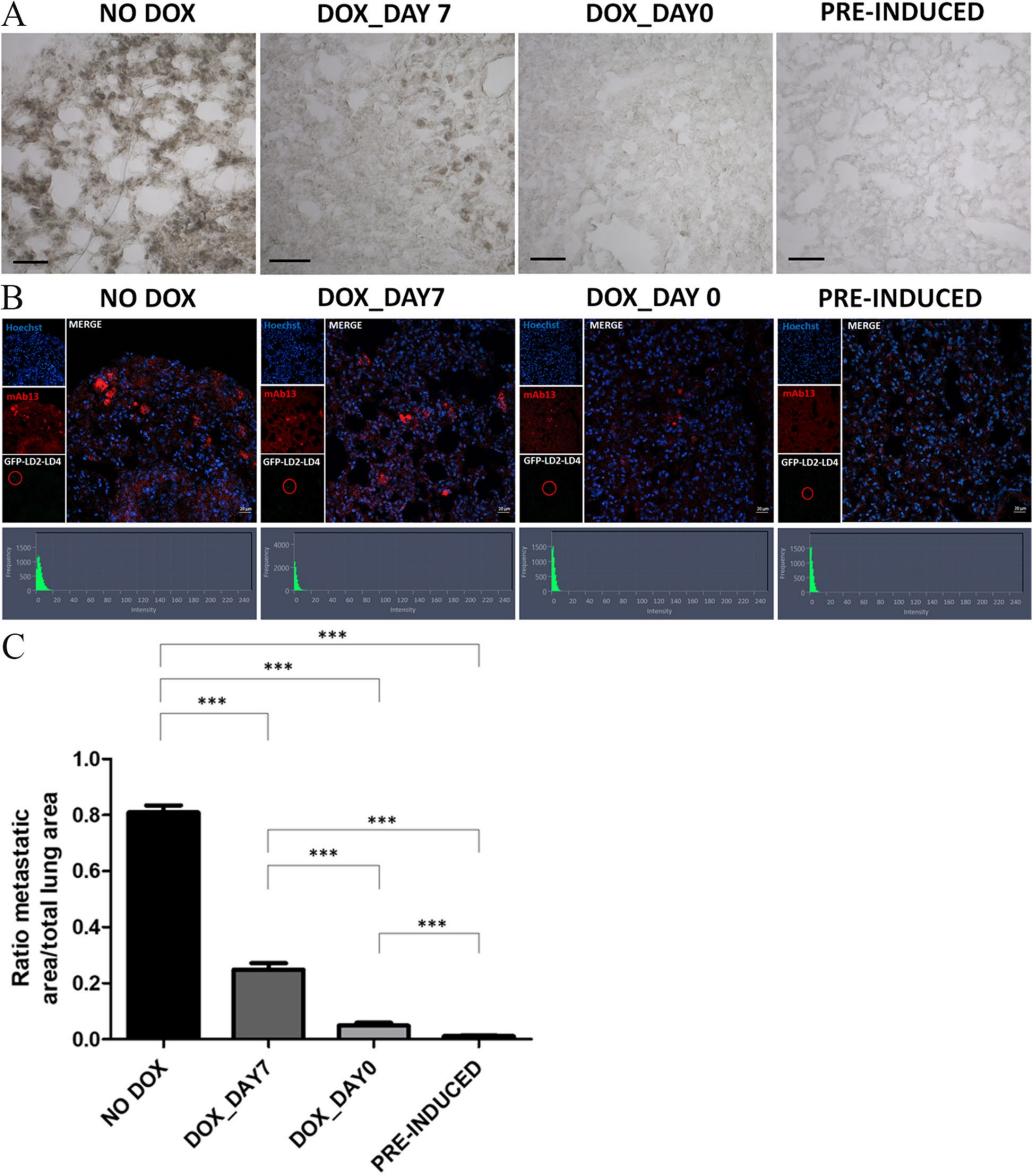
LD2-LD4 expression dramatically inhibits metastasis in vivo. **A** Representative stereoscope images of frozen lung sections, processed for chromogenic immunohistochemistry demonstrating lung metastasis, using an anti-human specific antibody, namely anti-mAb13, for the detection of LM2 cells. Scale bars: 100 μm. **B** Representative confocal images of frozen lung sections processed for fluorescent immunohistochemistry demonstrating lung metastasis, using mAb13 for the detection of LM2 cells. Cell nuclei were visualized using Hoechst staining. ZEN graphs show GFP intensity for each selected ROI defined by a red circle. Scale bars: 20 μm. **C** Quantification of lung metastasis of control and treated mice, using two-tailed unpaired t test. The mean ratio of metastatic area/total lung area of non-treated mice (NO DOX) is 0.8093 ± 0.02517, for the DOX_DAY7 group is 0.2488 ± 0.02269, for the DOX_DAY0 group is 0.04862 ± 0.01022 and for PRE-INDUCED group is 0.01097 ± 0.002382. SEM is represented by error bars. *N* = 13 sections per group, ***P ≤ 0.0001

**Fig. 11:**
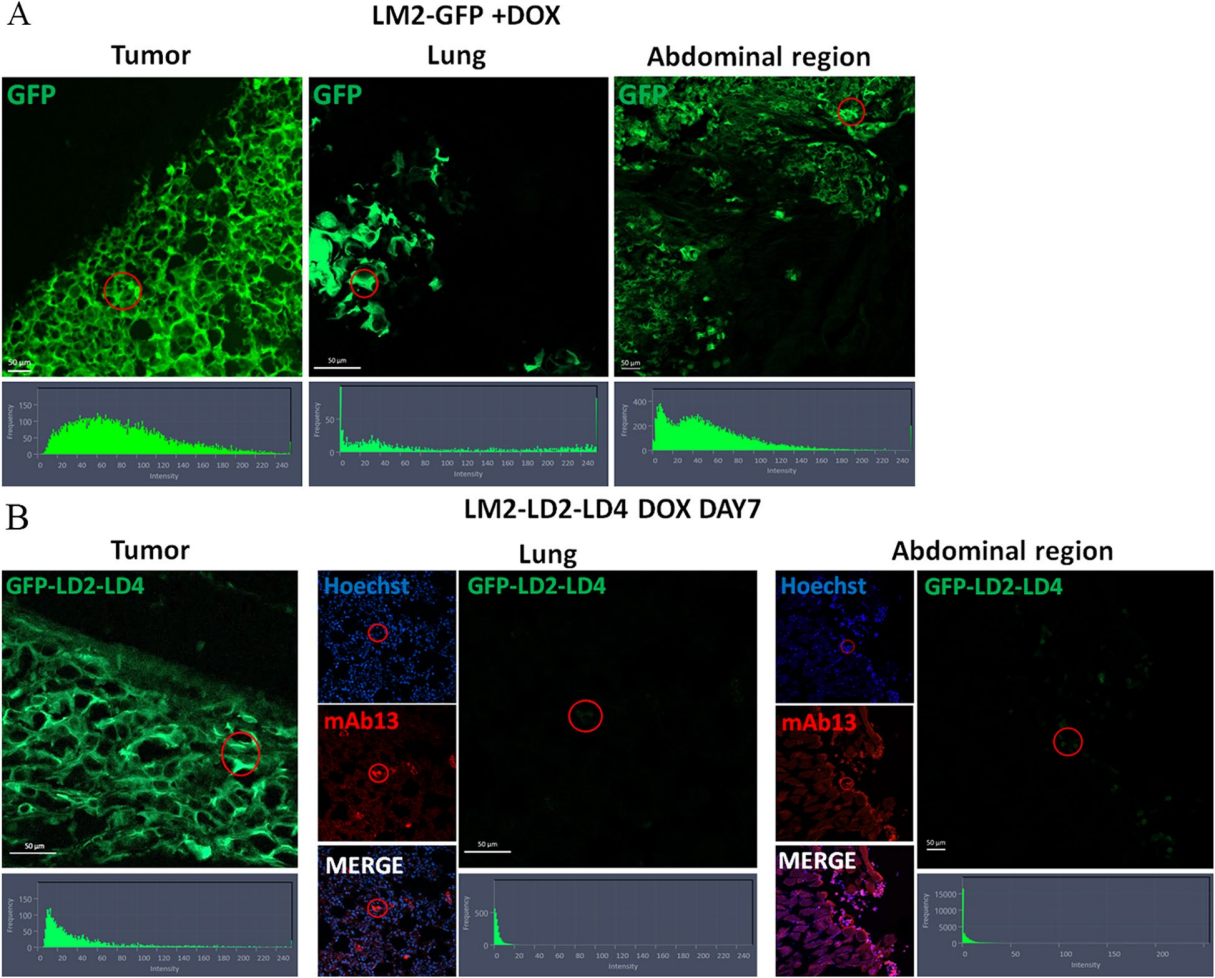
LM2 cells metastasize only if they switch off LD2-LD4 expression. **A**, **B** Representative confocal images of frozen sections processed for fluorescent immunohistochemistry. Tumors, lungs, and abdominal metastatic foci were isolated 9 weeks following orthotopic injection of 2 × 10^6^ LM2-GFP cells (**A**) or LM2-LD2-LD4 cells (DOX DAY7 group) (**B**) in the mammary fat pad of NOD/SCID mice. mAb13 was used for the detection of metastatic LM2 cells in doxycycline-treated mice. ZEN graphs show GFP intensity for each selected ROI defined by a red circle. Scale bars: 50 μm

We then evaluated the in vivo efficacy of LD2-LD4 against metastasis in the above-described mice groups. LD2-LD4 expression clearly suppressed metastasis of LM2 cells in vivo, nearly abolishing the appearance of metastatic tumors, in an expression time-dependent manner; the earlier the time of induction the greater the inhibition of lung metastasis and the fewer the metastatic foci detected, similarly to orthotopic tumor growth (Figs. 9 and 10). More specifically, mice injected with pre-induced LM2-LD2-LD4 cells or induced on Day 0 (prophylactic treatment) failed to develop metastasis in the abdominal region and metastatic foci in the lungs were non-existent or minimal, upon macroscopic observation (Fig. 9B, E, F), whereas mice injected with LM2-LD2-LD4 cells induced on Day 7 (therapeutic treatment) were found to have metastasis in the abdominal region, and metastatic foci in the lungs. These were fewer than the control group, however clearly present unlike the pre-induced group. This result was confirmed by quantification performed on multiple lung sections following chromogenic immunohistochemistry (Fig. 10A), demonstrating significantly reduced pulmonary metastasis in all three groups of mice injected with LM2 cells expressing LD2-LD4 (either pre-induced or induced on Day 0 or Day 7) in comparison to control mice (therapeutic treatment: 3.3-fold reduced pulmonary metastasis compared to control, *P* < 0.0001; prophylactic treatment: 16.6-fold reduced pulmonary metastasis compared to control, *P* < 0.0001; pre-induction: 73.8-fold reduced pulmonary metastasis compared to control, *P* < 0.0001) (Fig. 10C). Interestingly, the three LD2-LD4-expressing groups also demonstrated significant differences between them (pre-induction had 4.4-fold less metastasis compared to prophylactic treatment, *P* = 0.0001, and 22.7-fold less metastasis compared to therapeutic treatment, *P* < 0.0001; prophylactic treatment had 5.1-fold less metastasis compared to therapeutic treatment, *P* < 0.0001) (Fig. 10C). Importantly, using fluorescent immunohistochemistry, we showed that metastatic foci (from both lungs and abdominal region) of mice expressing LD2-LD4 contained mAb13 + GFP- cells (mAb13 serves as a marker indicating cells of human origin) (Figs. 10B and 11B, Additional file 1: Fig. S5C), while the respective orthotopic tumors contained mAb13 + GFP + cells (Fig. 11B), unlike metastatic foci and orthotopic tumors from the control GFP groups which both contained only mAb13 + GFP + cells (Fig. 11A, Additional file 1: Fig. S5A-D). The loss of GFP expression from the metastatic foci from LD2-LD4 induced mice shows that LD2-LD4 expression dramatically inhibits metastasis, and its expression must be turned off for metastasis to take place. It also suggests that the differences observed between pre-induced, therapeutic and prophylactic treatments in terms of impact on metastasis likely stem from greater expansion of the tumor cells in the therapeutic treatment group prior to LD2-LD4 expression, which allows a larger number of tumor cells to shut down LD2-LD4 expression. These results clearly show that expression of LD2-LD4 severely impairs the in vivo ability of MDA231-LM2-4175 tumor cells to grow orthotopically and metastasize. This outcome provides strong support for the potential of LD2-LD4 as a promising therapeutic approach against cancer, thus raising the possibility for the design and generation of effective small-molecule dual FAK/PYK2 inhibitors to prevent metastasis.

### A single LD motif can displace FAK and PYK2 from focal adhesions

**Fig. 12:**
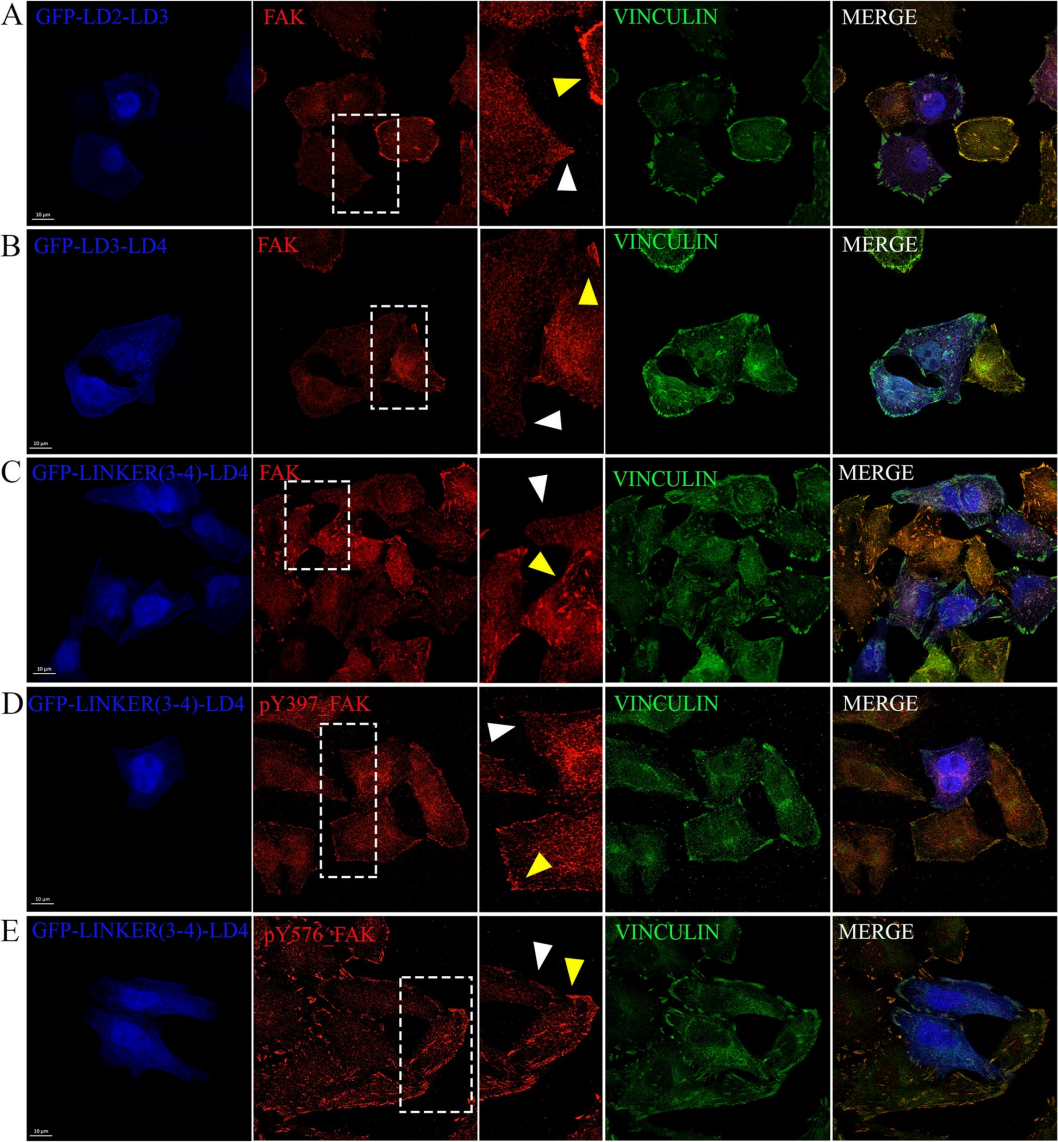
A single LD motif with increased affinity for the FAT domain displaces FAK from FAs and inhibits its acivity. Representative confocal images of HeLa cells transiently transfected with (**A**) GFP-LD2-LD3, (**B**) GFP-LD3-LD4, or (**C**-**E**) GFP-Linker(3–4)-LD4 peptide, immunostained for FAK (**A**-**C**), Vinculin (**A**-**E**), pFAK Y397 (**D**), and pFAK Y576 (**E**). Yellow arrows are pointing FAs of control cells and white arrows are pointing FAs of expressors. Dashed white rectangles indicate zoomed-in areas, shown in the middle column. Scale bars: 10 μm

**Fig. 13:**
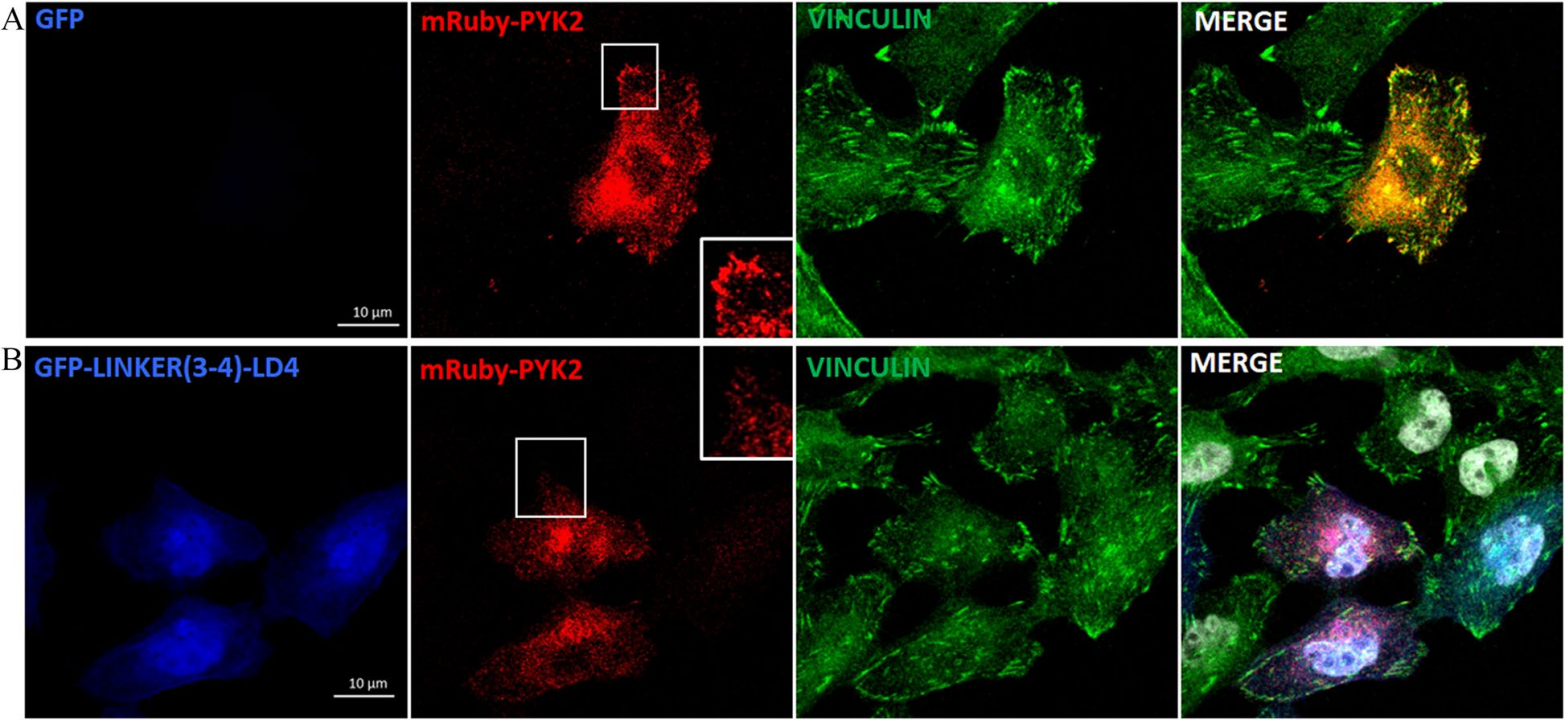
Linker(3–4)-LD4 peptide is effective in displacing PYK2 from FAs. Representative confocal images of HeLa cells transiently transfected with mRuby-PYK2 only (**A**) or co-transfected with GFP-Linker(3–4)-LD4 and mRuby-PYK2 (**B**), immunostained for Vinculin. White rectangles indicate zoomed-in areas. Scale bars: 10 μm

The cooperative binding of paxillin's LD2 and LD4 motifs to the FAT domain of FAK is crucial for forming a stable complex, with both motifs required to engage simultaneously for effective interaction [47]. The individual binding affinities of LD2 and LD4 motifs to the FAT domain are relatively weak. However, when both LD motifs bind simultaneously, they form a more stable and higher-affinity complex [48, 49]. We have previously shown that the LDs as monomers fail to displace FAK from focal adhesions [31] (Additional file 1: Fig. S10), while as dimers (LD2-LD2 and LD4-LD4) can effectively displace FAK [31], suggesting that a single molecule with sufficiently high affinity for a HP could potentially be used to target both HPs on the FAT domain. Mimicking the cooperative binding of LD2 and LD4 with a single small molecule presents a significant challenge given that the binding sites for LD2 and LD4 on the FAT domain are located on opposite sides, requiring a molecule with a large span to bridge the distance. This is difficult to achieve with conventional small molecules. We thus set out to ask if a single LD motif with sufficiently high affinity could mimic LD dimers. Given evidence that the linker regions have been shown to contribute to the affinity of the LD motif interactions with the FAT domain [50] and that LD3 does not bind the HPs [51, 52], we initially generated LD2-LD3 and LD3-LD4 constructs to examine if any of these could effectively achieve FAK displacement. Both constructs were able to displace FAK (Fig. 12A, B) suggesting that the linker regions do confer increased affinity to LD2 and LD4, and that a single LD could potentially be used to displace FAK from FAs. Despite evidence that LD3 does not bind the HPs when LD2 and LD4 are present [51, 52], it is possible that when only LD2 or only LD4 is present it does. To confirm that a single LD motif is sufficient we went ahead and deleted the LD3 motif from the LD3-LD4 construct and generated Linker(3–4)-LD4 construct. Although the affinity of LD4 for the HPs is lower than that of LD2, we opted for Linker(3–4)-LD4, since the Linker(3–4) is smaller than the one between LD2 and LD3 leading to the generation of a smaller peptide (LD2-LD3 linker: 51aa, LD3-LD4 linker: 28aa). As shown in Fig. 12, this construct can effectively displace FAK from FAs (Fig. 12C) and inhibits the phosphorylation of both FAK Tyrosine 397 and 576 (Fig. 12D and E). Linker(3–4)-LD4 is also effectively displacing PYK2 similarly to FAK (Fig. 13), and inhibits the autophosphorylation of PYK2 on Tyrosine 402 at focal adhesions (Additional file 1: Fig. S11 A). In addition, its expression significantly inhibits MDA-MB-231 cell migration (*P* < 0.0001) and LM2 cell invasion in 3D-spheroid assays (*P* < 0.0001) (Fig. S11B, C, D). These data show that a single LD motif with high affinity can displace both FAK and PYK2 from FAs and inhibit their activity. Linker(3–4)-LD4 is the shortest polypeptide we have tested (46aa) that can effectively inhibit both FAK-related kinases and well within the size range of solid phase peptide synthesis (SPPS). It also suggests that a small molecule with high affinity for the HPs could be developed and effectively displace FAK and PYK2.

## Discussion

FAK and its closely related paralogue PYK2 are key regulators of tumorigenesis, tumor progression and metastasis, since they both regulate tumor cell survival, proliferation, spreading, migration, invasion, and chemo-resistance, and have been previously associated with poor prognosis [2, 18]. In the present study, we evaluated a novel strategy for the inhibition of both FAK and PYK2 in a xenograft mouse model, as a therapeutic approach against tumor progression and metastasis. The strategy relies on the use of a short peptide, containing the LD2 and LD4 motifs of Paxillin (LD2-LD4 peptide), that competes with interactions taking place between endogenous LD motifs and hydrophobic pockets on the kinases’ FAT domains, thus leading to the displacement of both kinases from FAs [31].

We have previously shown that expression of LD2-LD4 not only led to a dose-dependent elimination of endogenous FAK from FAs, but also inhibited FAK/Src signaling at FAs and resulted in reduced phosphorylation of downstream FAK targets [31]. Therefore, expression of LD2-LD4 was shown to effectively interfere with interactions taking place at the HPs of the FAT domain of FAK and was found to be sufficient to block both FA-related kinase and scaffolding functions of FAK, known to promote cancer progression and metastasis [25, 26, 31]. Intriguingly, it was shown that the LD2 and LD4 motifs of Paxillin also interact with the FAT domain of PYK2 [50, 53]. FAK and PYK2 are involved in overlapping signaling pathways, implicated in the development of tumor malignancy; PYK2 is upregulated upon deletion or inhibition of FAK to compensate for the loss of FAK activity [17, 21, 22]. More specifically, when FAK is knocked out, PYK2 increases RHO GTPase activation, facilitates angiogenesis and regulates macrophage motility and tumorigenic outgrowth [1, 10, 22]. In addition, FAK-specific inhibitors that are not dual inhibitors, have been shown to enhance PYK2 tyrosine phosphorylation in endothelial cells [10], indicating that PYK2 activity could potentially weaken the impact/effect of such inhibitors. In this work, we show that expression of the LD2-LD4 peptide efficiently targets and completely displaces PYK2 from FAs, even though Paxillin is not PYK2’s native binding partner and their interaction has been reported to be unstable [50, 53]. This result may be supported by recent molecular dynamics simulations of LD motif recognition by FAT domains, using short (12-residue) peptide segments, that revealed a preference of Paxillin LD motifs for PYK2 over FAK [54]. Physiologically, this might not be observed, since FAT domains do not interact with isolated LD motifs but form complexes with full length Paxillin, which includes LD-motif flanking regions; these may alter binding affinities and lead to preferential association with FAK rather than PYK2. However, our approach employs a simple peptide which does not contain flanking regions, thus it should be able to bind the FAT domain of PYK2 with an equal affinity as that of FAK, if not better.

In comparison to FAK, PYK2 is not as strongly localized at FAs in most cell types [18]. This observation is most likely not due to a lower affinity for Paxillin but rather due to the ability of the FAK FERM domain to bind PIP2 and increase local concentration of FAK at FAs [55]. This is further supported by work showing that swapping the FAK and PYK2 FERM domains leads to increased FA localization of PYK2 and reduced FA localization of FAK [56]. Thus, given the reduced targeting/localization of PYK2 at FAs, it is anticipated that a lower concentration of LD2-LD4 peptide would be sufficient for PYK2 displacement from these structures, than that required to displace FAK.

Both FAK and PYK2 also bind the LD motifs of the Paxillin family members Hic- 5 (Hydrogen-peroxide Inducible Clone-5) and Leupaxin [50, 57, 58]. Hic-5 and Leupaxin are highly expressed in various cancers and have been shown to promote progression and invasion of cancer cells [59]. Therefore, our approach allows effective competition with multiple LD-containing binding partners of FAK and PYK2, an important consideration when trying to design targeted inhibitors.

The efficient displacement of both FAK and PYK2 from FAs, due to the expression of LD2-LD4 peptide, results in a dramatic reduction in the phosphorylation levels of Y397 and Y402 on FAK and PYK2 respectively, key residues for the kinases’ activation and downstream signaling [60]. Thus, even though PYK2 is not strongly targeted to FAs in most cells [26], our data show that LD2-LD4 can both displace the protein from FAs and inhibit its activation, thus providing a unique strategy of dual inhibition, that can concomitantly block both enzymatic and scaffolding functions of both kinases.

Expression of LD2-LD4 lead to the displacement of FAK and PYK2 from FAs in various cell lines, but the overall FA composition was not affected. Major FA proteins such as Talin, Paxillin, Vinculin and ILK remained associated with FAs, suggesting that the peptide specifically disrupted the interaction of Paxillin with the intended targets. Talin does not rely on its interaction with the LD2 motif of Paxillin for its recruitment to mature FAs [61], however its recruitment to nascent FAs depends on FAK and specifically on a direct interaction of the Talin FERM F3 lobe with the FAT domain of FAK [62]. This interaction is critical for cell migration, since FAK mutants harboring a mutation (E1015 A) leading to disruption of the FAK-Talin interaction, fail to rescue migration defects of FAK null cells [62]. This raises the possibility that migration and invasion defects observed in LD2-LD4 expressing tumor cells may, at least in part, stem from the elimination of Talin from nascent adhesions. The binding site on the FAK FAT domain, which interacts with the Talin FERM domain, is at a considerable distance from the HPs bound by the Paxillin LDs [47] presumably allowing concomitant binding of the FAT domain of FAK to both the LDs and the Talin FERM domain and subsequent Talin recruitment to nascent FAs. A FAK kinase inhibitor, would most certainly be incapable of affecting this critical scaffolding role of FAK, since none of the interactions involved are dependent on FAK’s enzymatic activity. Therefore, this could explain the drastic inhibition of metastasis upon LD2-LD4 expression, compared to the impact of kinase inhibition, and warrants further study. Moreover, it further highlights the advantages of the proposed approach compared to using kinase inhibitors [63].

Collectively, the results of the current preclinical study show that the LD2-LD4 peptide exerts promising antitumor activity and that FAK/PYK2 displacement from FAs constitutes a new promising inhibitory strategy. Particularly, LD2-LD4 expression suppressed tumor growth significantly, and nearly eliminated metastasis. Control group mice developed the largest tumors, compared to the LD2-LD4-induced groups; specifically, in the group in which LD2-LD4 expression was pre-induced, tumor initiation was remarkably inhibited, even 5 weeks post-injection. Even more importantly, LD2-LD4 expression dramatically suppressed the development of metastatic nodules in both the lungs and the abdominal region. Notably, our data show that tumor cells are unable to metastasize unless LD2-LD4 expression is switched off. This is more pronounced when induction was initiated 7 days after the tumor cells were introduced in the mice suggesting that the slow buildup of LD peptide allows the selection of cells that shut down transgene expression. This result provides strong support that targeting the FAK/PYK2 hydrophobic pockets is a strategy that could be highly effective and could lead the development of novel anti-metastatic agents. Importantly, we have previously shown that an LD2 dimer or an LD4 dimer can also displace FAK from FAs suggesting that it would be possible to target both HPs with a single molecule [31]. We now show that an LD monomer can displace both FAK and PYK2 from FAs. We are therefore currently exploring this possibility using peptidomimetic compounds designed to mimic the LD motifs, in an effort to come a step closer in the design of an effective anti-tumor and anti-metastatic agent, that could pave the way towards clinical application of this strategy.

## Conclusion

Displacement from focal adhesions is a novel strategy that uniquely blocks both enzymatic and scaffolding functions of FAK and PYK2. It dramatically suppresses metastasis and could allow the development of more effective small molecule dual FAK/PYK2 inhibitors.

## Supplementary Information

Additional file 1: Fig. S1. LD2-LD4 effectively displaces PYK2 from FAs in FAK null cells. Fig. S2. LD2-LD4 effectively displaces both PYK2 and FAK from FAs in U-118 MG cells. Fig. S3. Doxycycline has a strong anti-proliferative effect on MDA-MB-231 cells. Fig. S4. Doxycycline does not inhibit orthotopic MDA231-LM2- 4175 tumor growth and metastatic spread. Fig. S5. LM2-GFP cells detected in the lungs and abdominal region of non-treated and doxycycline-treated mice. Fig. S6. LD2-LD4 displaces PYK2 from focal adhesions. Fig. S7. Displacement of FAK and PYK2 from FAs by LD2-LD4 expression does not affect overall FA composition. Fig. S8. LD2-LD4 inhibits FAK kinase-dependent functions and downstream signaling in LM2 cells. Fig. S9. LD2-LD4 inhibits cell migration of SUM149 and SUM159 cells. Fig. S10. Single LD motifs do not displace FAK from focal adhesions. Fig. S11. Linker(3-4)-LD4 peptide expression inhibits tumor cell migration and invasion.

## Data Availability

No datasets were generated or analysed during the current study.

## Abbreviations

FAs: Focal adhesions
FAK: Focal Adhesion Kinase
PYK2: Proline-rich tyrosine kinase 2
FAT: Focal Adhesion Targeting
HPs: Hydrophobic Pockets
GFP: Green Fluorescent Protein
SH2: Src Homology 2
PROTAC: Proteolysis Targeting Chimeric Molecule
IP: Immunoprecipitation
2D: 2-Dimensional
3D: 3-Dimensional
ROI: Region of interest

## References

[1] Yoon H, Dehart JP, Murphy JM, Lim S-TS. Understanding the roles of FAK in cancer: inhibitors, genetic models, and new insights. J Histochem Cytochem. 2015;63(2):114–28. 25380750 10.1369/0022155414561498 PMC4305513

[2] Pang X-J, Liu X-J, Liu Y, Liu W-B, Li Y-R, Yu G-X, et al. Drug Discovery Targeting Focal Adhesion Kinase (FAK) as a Promising Cancer Therapy. Molecules. 2021;26(14):4250. 34299525 10.3390/molecules26144250 PMC8308130

[3] Brown MC, Perrotta JA, Turner CE. Identification of LIM3 as the principal determinant of paxillin focal adhesion localization and characterization of a novel motif on paxillin directing vinculin and focal adhesion kinase binding. J Cell Biol. 1996;135(4):1109–23. 8922390 10.1083/jcb.135.4.1109 PMC2133378

[4] Schaller MD. Cellular functions of FAK kinases: insight into molecular mechanisms and novel functions. J Cell Sci. 2010;123(7):1007–13. 20332118 10.1242/jcs.045112

[5] Acebrón I, Righetto RD, Schoenherr C, de Buhr S, Redondo P, Culley J, et al. Structural basis of Focal Adhesion Kinase activation on lipid membranes. EMBO J. 2020;39(19):e104743. 32779739 10.15252/embj.2020104743 PMC7527928

[6] Cary LA, Han DC, Polte TR, Hanks SK, Guan J-L. Identification of p130Cas as a Mediator of Focal Adhesion Kinase–promoted Cell Migration. J Cell Biol. 1998;140(1):211–21. 9425168 10.1083/jcb.140.1.211 PMC2132604

[7] Frame MC, Patel H, Serrels B, Lietha D, Eck MJ. The FERM domain: organizing the structure and function of FAK. Nat Rev Mol Cell Biol. 2010;11(11):802–14. 20966971 10.1038/nrm2996

[8] Lim S-T, Chen XL, Lim Y, Hanson DA, Vo T-T, Howerton K, et al. Nuclear FAK Promotes Cell Proliferation and Survival through FERM-Enhanced p53 Degradation. Molec Cell. 2008;29(1):9–22. 18206965 10.1016/j.molcel.2007.11.031 PMC2234035

[9] Serrels A, Lund T, Serrels B, Byron A, McPherson RC, et al. Nuclear FAK Controls Chemokine Transcription, Tregs, and Evasion of Anti-tumor Immunity. Cell. 2015;163(1):160–73. 26406376 10.1016/j.cell.2015.09.001 PMC4597190

[10] Sulzmaier FJ, Jean C, Schlaepfer DD. FAK in cancer: mechanistic findings and clinical applications. Nat Rev Cancer. 2014;14(9):598–610. 25098269 10.1038/nrc3792 PMC4365862

[11] Lee BY, Timpson P, Horvath LG, Daly RJ. FAK signaling in human cancer as a target for therapeutics. Pharmacol Ther. 2015;146:132–49. 25316657 10.1016/j.pharmthera.2014.10.001

[12] Dawson JC, Serrels A, Stupack DG, Schlaepfer DD, Frame MC. Targeting FAK in anticancer combination therapies. Nat Rev Cancer. 2021;21(5):313–24. 33731845 10.1038/s41568-021-00340-6 PMC8276817

[13] Agochiya M, Brunton VG, Owens DW, Parkinson EK, Paraskeva C, Keith WN, et al. Increased dosage and amplification of the focal adhesion kinase gene in human cancer cells. Oncogene. 1999;18(41):5646–53. 10523844 10.1038/sj.onc.1202957

[14] Kaveh F, Baumbusch LO, Nebdal D, Børresen-Dale A-L, Lingjærde OC, Edvardsen H, et al. A systematic comparison of copy number alterations in four types of female cancer. BMC Cancer. 2016;16(1):913. 27876019 10.1186/s12885-016-2899-4 PMC5120489

[15] Golubovskaya VM. Focal adhesion kinase as a cancer therapy target. Anticancer Agents Med Chem. 2010;10(10):735–41. 21214510 10.2174/187152010794728648 PMC3267551

[16] Zhu X, Bao Y, Guo Y, Yang W. Proline-Rich Protein Tyrosine Kinase 2 in Inflammation and Cancer. Cancers. 2018;10(5):139. 29738483 10.3390/cancers10050139 PMC5977112

[17] Walkiewicz KW, Girault J-A, Arold ST. How to awaken your nanomachines: Site-specific activation of focal adhesion kinases through ligand interactions. Prog Biophys Mol Biol. 2015;119(1):60–71. 26093249 10.1016/j.pbiomolbio.2015.06.001

[18] Naser R, Aldehaiman A, Díaz-Galicia E, Arold S. Endogenous control mechanisms of FAK and PYK2 and their relevance to cancer development. Cancers. 2018;10(6):196. 29891810 10.3390/cancers10060196 PMC6025627

[19] Lulo J, Yuzawa S, Schlessinger J. Crystal structures of free and ligand-bound focal adhesion targeting domain of Pyk2. Biochem Biophys Res Commun. 2009;383(3):347–52. 19358827 10.1016/j.bbrc.2009.04.011

[20] Gao C, Chen G, Kuan S-F, Zhang DH, Schlaepfer DD, Hu J. FAK/PYK2 promotes the Wnt/β-catenin pathway and intestinal tumorigenesis by phosphorylating GSK3β. Elife. 2015;4:e10072. 26274564 10.7554/eLife.10072 PMC4558782

[21] Weis SM, Lim S-T, Lutu-Fuga K, Barnes L, Chen X, Göthert JR, et al. Compensatory role for Pyk2 during angiogenesis in adult mice lacking endothelial cell FAK. J Cell Biol. 2008;181(1):43–50. 18391070 10.1083/jcb.200710038 PMC2287283

[22] Fan H, Guan J-L. Compensatory Function of Pyk2 Protein in the Promotion of Focal Adhesion Kinase (FAK)-null Mammary Cancer Stem Cell Tumorigenicity and Metastatic Activity. J Biol Chem. 2011;286(21):18573–82. 21471206 10.1074/jbc.M110.200717 PMC3099673

[23] Mohanty A, Pharaon RR, Nam A, Salgia S, Kulkarni P, Massarelli E. FAK-targeted and combination therapies for the treatment of cancer: an overview of phase I and II clinical trials. Expert Opin Investig Drugs. 2020;29(4):399–409. 32178538 10.1080/13543784.2020.1740680

[24] Quispe PA, Lavecchia MJ, León IE. Focal adhesion kinase inhibitors in the treatment of solid tumors: Preclinical and clinical evidence. Drug Discov Today. 2021;27(2):664–74. 34856395 10.1016/j.drudis.2021.11.025

[25] Cance WG, Kurenova E, Marlowe T, Golubovskaya VM. Disrupting the scaffold to improve focal adhesion kinase-targeted cancer therapeutics. Sci Signal. 2013;6(268):e10. 10.1126/scisignal.2004021 PMC3693475 23532331

[26] Tai Y-L, Chen L-C, Shen T-L. Emerging Roles of Focal Adhesion Kinase in Cancer. Biomed Res Int. 2015;2015:1–13. 10.1155/2015/690690 PMC4396139 25918719

[27] Law RV, Rodrigues O, Chung C, Bantscheff M, Buda K, Dai H, et al. Discovery and Characterisation of Highly Cooperative FAK-Degrading PROTACs. Angew Chem Int Ed Engl. 2021;60(43):23327–34. 34416073 10.1002/anie.202109237

[28] Khan S, He Y, Zhang X, Yuan Y, Pu S, Kong Q, et al. PROteolysis TArgeting Chimeras (PROTACs) as emerging anticancer therapeutics. Oncogene. 2020;39(26):4909–24. 32475992 10.1038/s41388-020-1336-y PMC7319888

[29] Liu Z, Hu M, Yang Y, Du C, Zhou H, Liu C, et al. An overview of PROTACs: a promising drug discovery paradigm. Mol Biomed. 2022;3(1):46. 36536188 10.1186/s43556-022-00112-0 PMC9763089

[30] Arold ST, Hoellerer MK, Noble MEM. The structural basis of localization and signaling by the focal adhesion targeting domain. Structure. 2002;10(3):319–27. 12005431 10.1016/s0969-2126(02)00717-7

[31] Antoniades I, Kyriakou M, Charalambous A, Kalalidou K, Christodoulou A, Christoforou M, et al. FAK displacement from focal adhesions: a promising strategy to target processes implicated in cancer progression and metastasis. Cell Commun Signal. 2021;19(1):3. 33413438 10.1186/s12964-020-00671-1 PMC7791867

[32] Sancho D, Nieto M, Llano M, Rodríguez-Fernández JL, Tejedor R, Avraham S, et al. The Tyrosine Kinase Pyk-2/Raftk Regulates Natural Killer (Nk) Cell Cytotoxic Response, and Is Translocated and Activated upon Specific Target Cell Recognition and Killing. J Cell Biol. 2020;149(6):1249–62. 10.1083/jcb.149.6.1249 PMC2175114 10851022

[33] Pereira TF, Levin G, DeOcesano-Pereira C, Caodaglio AS, Fujita A, Tonso A, et al. Fluorescence-based method is more accurate than counting-based methods for plotting growth curves of adherent cells. BMC Res Notes. 2020;13(1):57. 32019595 10.1186/s13104-020-4914-8 PMC7001368

[34] Kuo J-C, Han X, Yates JR, Waterman CM. Isolation of Focal Adhesion Proteins for Biochemical and Proteomic Analysis. Methods Mol Biol. 2011;757:297–323. 10.1007/978-1-61779-166-6_19 PMC4158431 21909920

[35] Skourides, P.A., Hadjigeorgiou, A. and Christodoulou, N. Tumor microenvironment mimicking device and method. WIPO. WO2023084483A1. 2023. Available at: https://patents.google.com/patent/WO2023084483A1/en?oq=WO2023084483A1.

[36] Gossen M, Freundlieb S, Bender G, Muller G, Hillen W, Bujard H. Transcriptional activation by tetracyclines in mammalian cells. Science. 1995;268(5218):1766–9. 7792603 10.1126/science.7792603

[37] Sieg DJ. Pyk2 and Src-family protein-tyrosine kinases compensate for the loss of FAK in fibronectin-stimulated signaling events but Pyk2 does not fully function to enhance FAK- cell migration. EMBO J. 1998;17(20):5933–47. 9774338 10.1093/emboj/17.20.5933 PMC1170921

[38] Mitra SK, Hanson DA, Schlaepfer DD. Focal adhesion kinase: in command and control of cell motility. Nat Rev Mol Cell Biol. 2005;6(1):56–68. 15688067 10.1038/nrm1549

[39] Zhao M, Finlay D, Zharkikh I, Vuori K. Novel Role of Src in Priming Pyk2 Phosphorylation. PLoS One. 2016;11(2):e0149231. 26866924 10.1371/journal.pone.0149231 PMC4750869

[40] Murphy JM, Park H, Lim S-TS. FAK and Pyk2 in disease. Front Biol. 2016;11(1):1–9.

[41] Contestabile A, Bonanomi D, Burgaya F, Girault J, Valtorta F. Localization of focal adhesion kinase isoforms in cells of the central nervous system. Int J Dev Neurosci. 2003;21(2):83–93. 12615084 10.1016/s0736-5748(02)00126-0

[42] Luo M, Guan J-L. Focal adhesion kinase: A prominent determinant in breast cancer initiation, progression and metastasis. Cancer Lett. 2010;289(2):127–39. 19643531 10.1016/j.canlet.2009.07.005 PMC2854647

[43] Al-Juboori SIK, Vadakekolathu J, Idri S, Wagner S, Zafeiris D, Pearson JRD, et al. PYK2 promotes HER2-positive breast cancer invasion. J Exp Clin Cancer Res. 2019;38(1):210. 31118051 10.1186/s13046-019-1221-0 PMC6532260

[44] Chen Y-F, Yang Y-N, Chu H-R, Huang T-Y, Wang S-H, Chen H-Y, et al. Role of integrin αvβ3 in doxycycline-induced anti-proliferation in breast cancer cells. Front Cell Dev Biol. 2022;10:829788. 35237605 10.3389/fcell.2022.829788 PMC8884148

[45] Selicharova I, Sanda M, Mladkova J, Ohri S, Vashishta A, Fusek M, et al. 2-DE analysis of breast cancer cell lines 1833 and 4175 with distinct metastatic organ-specific potentials: Comparison with parental cell line MDA-MB-231. Oncol Rep. 2008;19(5):1237–44. 18425382

[46] Khan SU, Xia Y, Goodale D, Schoettle G, Allan AL. Lung-Derived selectins enhance metastatic behavior of triple negative breast cancer cells. Biomedicines. 2021;9(11):1580. 34829810 10.3390/biomedicines9111580 PMC8615792

[47] Bertolucci CM, Guibao CD, Zheng J. Structural features of the focal adhesion kinase-paxillin complex give insight into the dynamics of focal adhesion assembly. Protein Sci. 2005;14(3):644–52. 15689512 10.1110/ps.041107205 PMC2279287

[48] Neerathilingam M, Bairy SG, Mysore S. Deciphering Mode of Action of Functionally Important Regions in the Intrinsically Disordered Paxillin (Residues 1–313) Using Its Interaction with FAT (Focal Adhesion Targeting Domain of Focal Adhesion Kinase). PLoS One. 2016;11(2):e0150153. 26928467 10.1371/journal.pone.0150153 PMC4771712

[49] Le Coq J, Acebrón I, Rodrigo Martin B, LópezNavajas P, Lietha D. New insights into FAK structure and function in focal adhesions. J Cell Sci. 2022;135(20):jcs259089. 36239192 10.1242/jcs.259089

[50] Vanarotti M, Finkelstein D, Guibao CD, Nourse A, Miller DJ, Zheng J. Structural Basis for the Interaction between Pyk2-FAT Domain and Leupaxin LD Repeats. Biochemistry. 2016;55(9):1332–45. 26866573 10.1021/acs.biochem.5b01274 PMC4843776

[51] Manetti ME, Geden S, Bott M, Sparrow N, Lambert S, Fernandez-Valle C. Stability of the tumor suppressor merlin depends on its ability to bind paxillin LD3 and associate with β1 integrin and actin at the plasma membrane. Biol Open. 2012;1(10):949–57. 23213372 10.1242/bio.20122121 PMC3507182

[52] Bhattacharya S, He Y, Chen Y, Mohanty A, Grishaev A, Kulkarni P, Ravi Salgia and Orban J. Conformational dynamics and multi-modal interaction of Paxillin with the Focal Adhesion Targeting Domain. bioRxiv. 2025 10.1126/sciadv.adt9936 PMC12175908 40532016

[53] Vanarotti M, Miller DJ, Guibao CD, Nourse A, Zheng J. Structural and Mechanistic Insights into the Interaction between Pyk2 and Paxillin LD Motifs. J Mol Biol. 2014;426(24):3985–4001. 25174335 10.1016/j.jmb.2014.08.014 PMC4267758

[54] Michael E, Polydorides S, Promponas VJ, Skourides PA, Archontis G. Recognition of LD motifs by the focal adhesion targeting domains of focal adhesion kinase and proline-rich tyrosine kinase 2-beta: Insights from molecular dynamics simulations. Proteins. 2020;89(1):29–52. 10.1002/prot.25992 32776636

[55] Goñi GM, Epifano C, Boskovic J, Camacho-Artacho M, Zhou J, Bronowska A, et al. Phosphatidylinositol 4,5-bisphosphate triggers activation of focal adhesion kinase by inducing clustering and conformational changes. Proc Natl Acad Sci U S A. 2014;111(31):E3177–86. 25049397 10.1073/pnas.1317022111 PMC4128148

[56] Dunty JM, Schaller MD. The N Termini of Focal Adhesion Kinase Family Members Regulate Substrate Phosphorylation, Localization, and Cell Morphology. J Biol Chem. 2002;277(47):45644–54. 12223467 10.1074/jbc.M201779200

[57] Nocula-Lugowska M, Lugowski M, Salgia R, Kossiakoff AA. Engineering Synthetic Antibody Inhibitors Specific for LD2 or LD4 Motifs of Paxillin. J Mol Biol. 2015;427(15):2532–47. 26087144 10.1016/j.jmb.2015.06.004 PMC4521624

[58] Sun CK, Ng KT, Lim ZX, Cheng Q, Lo CM, Poon RT, Man K, Wong N, Fan ST. Proline-Rich Tyrosine Kinase 2 (Pyk2) Promotes Cell Motility of Hepatocellular Carcinoma through Induction of Epithelial to Mesenchymal Transition. PLoS ONE. 2011;6(4):e18878–e18878. 21533080 10.1371/journal.pone.0018878 PMC3080371

[59] Deakin NO, Pignatelli J, Turner CE. Diverse roles for the Paxillin family of proteins in cancer. Genes Cancer. 2012;3(5–6):362–70. 23226574 10.1177/1947601912458582 PMC3513785

[60] Nakamura K, Yano H, Schaefer E, Sabe H. Different modes and qualities of tyrosine phosphorylation of Fak and Pyk2 during epithelial-mesenchymal transdifferentiation and cell migration: analysis of specific phosphorylation events using site-directed antibodies. Oncogene. 2001;20(21):2626–35. 11420674 10.1038/sj.onc.1204359

[61] Aziz AUR, Deng S, Jin Y, Li N, Zhang Z, Yu X, et al. The explorations of dynamic interactions of paxillin at the focal adhesions. Biochim Biophys Acta Proteins Proteom. 2022;1870(10):140825–140825. 35926716 10.1016/j.bbapap.2022.140825

[62] Lawson C, Lim S-T, Uryu S, Chen XL, Calderwood DA, Schlaepfer DD. FAK promotes recruitment of talin to nascent adhesions to control cell motility. J Cell Biol. 2012;196(3):387–387. 10.1083/jcb.201108078 PMC3265949 22270917

[63] Walsh CJ, Tanjoni I, Uryu S, Tomar A, Nam J-O, Luo H, et al. Oral delivery of PND-1186 FAK inhibitor decreases tumor growth and spontaneous breast to lung metastasis in pre-clinical models. Cancer Biol Ther. 2010;9(10):778–90. 20234193 10.4161/cbt.9.10.11433 PMC2933309

