# Supplementary material for: Targeting the hydrophobic pockets of FAK/PYK2 FAT domain: a highly effective inhibitory strategy suppressing tumor growth and eliminating metastasis
Source: Cell Commun Signal. 2025 May 19;23:231. doi: 10.1186/s12964-025-02203-1 (PMC12087225; doi:10.1186/s12964-025-02203-1)
Supplement: Supplementary file 1 — Additional file 1: Fig. S1. LD2-LD4 effectively displaces PYK2 from FAs in FAK null cells. Fig. S2. LD2-LD4 effectively displaces both PYK2 and FAK from FAs in U-118 MG cells. Fig. S3. Doxycycline has a strong anti-proliferative effect on MDA-MB-231 cells. Fig. S4. Doxycycline does not inhibit orthotopic MDA231-LM2- 4175 tumor growth and metastatic spread. Fig. S5. LM2-GFP cells detected in the lungs and abdominal region of non-treated and doxycycline-treated mice. Fig. S6. LD2-LD4 displaces PYK2 from focal adhesions. Fig. S7. Displacement of FAK and PYK2 from FAs by LD2-LD4 expression does not affect overall FA composition. Fig. S8. LD2-LD4 inhibits FAK kinase-dependent functions and downstream signaling in LM2 cells. Fig. S9. LD2-LD4 inhibits cell migration of SUM149 and SUM159 cells. Fig. S10. Single LD motifs do not displace FAK from focal adhesions. Fig. S11. Linker(3-4)-LD4 peptide expression inhibits tumor cell migration and invasion. [file 12964_2025_2203_MOESM1_ESM.docx]

Additional File 1

**Targeting the Hydrophobic Pockets of FAK/PYK2 FAT domain: a highly effective inhibitory strategy suppressing tumor growth and eliminating metastasis**

Maria Christoforou^1^, Anna Charalambous^1^, Dimitrios Sfakianakis^1^, Paris Alexander Skourides^1*^

**Supplementary Figures**

**Fig. S1.**


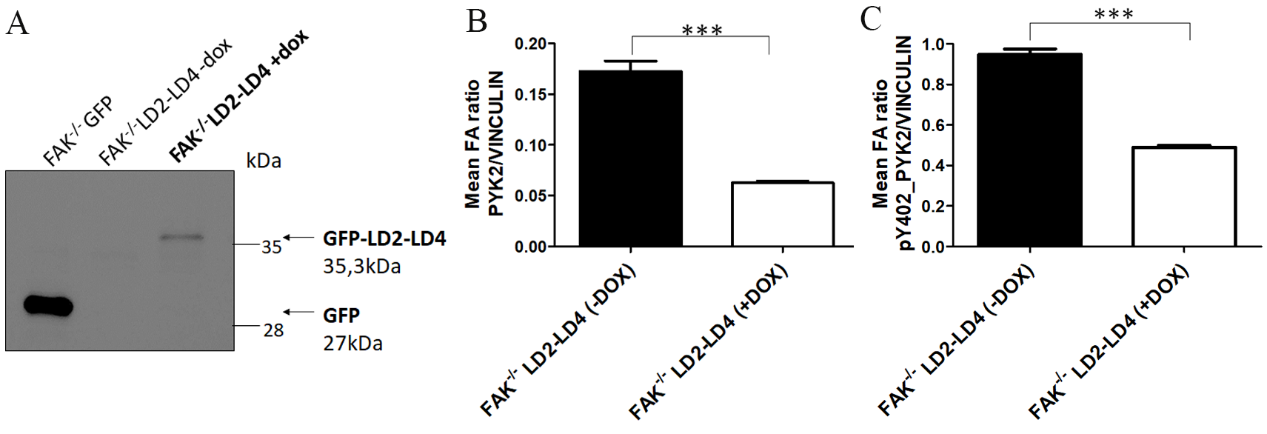


**Fig. S1.** **LD2-LD4 effectively displaces PYK2 from FAs in FAK null cells. (A)** Western Blot showing expression of GFP in FAK null cells stably expressing GFP, or inducibly expressing GFP-LD2-LD4, using an anti-GFP antibody. **(B)** Quantification of PYK2/Vinculin mean intensity ratio at FAs of FAK null LD2-LD4 cells (expressors and non-expressors) using two-tailed unpaired t test. The mean ratio of non-expressors is 0.1726 ± 0.01024 (N=410 FAs from 18 cells) and that of expressors is 0.06256 ± 0.001838 (N=390 FAs from 17 cells). SEM is represented by error bars. ***: P value < 0.0001. **(C)** Quantification of pY402_PYK2/Vinculin mean intensity ratio at FAs of FAK null LD2-LD4 stable cells (expressors and non-expressors) using two-tailed unpaired t test. The mean ratio of non-expressors is 0.9470 ± 0.02745 (N=342 FAs from 18 cells) and for expressors is 0.4895 ± 0.01050 N=376 FAs from 17 cells). SEM is represented by error bars. ***: P value < 0.0001.

**Fig. S2.**

**
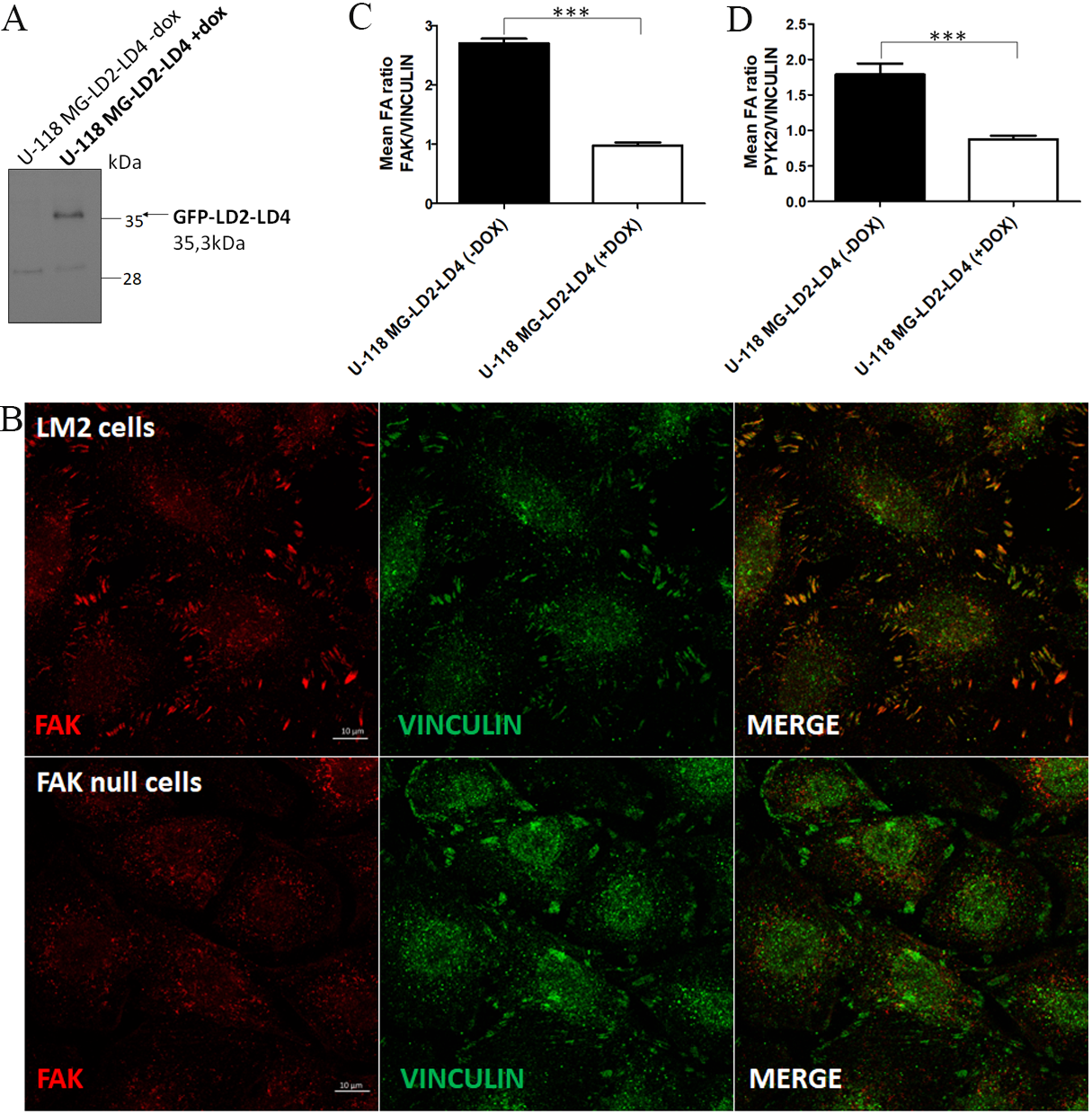
**

**Fig. S2.** **LD2-LD4 effectively displaces both PYK2 and FAK from FAs in U-118 MG cells. (A)** Western Blot showing GFP expression upon induction of LD2-LD4 in U-118 MG cells, using an anti-GFP antibody. **(B)** Representative confocal images of LM2 (upper lane) and FAK null cells (lower lane) immunostained for FAK and Vinculin. Scale bars: 10μm. **(C-D)** Quantification of **(C)** FAK/Vinculin (mean ratio is 2.699 ± 0.07718 in control cells and 0.9828 ± 0.05050 in LD2-LD4 expressors) and **(D)** PYK2/Vinculin (mean ratio is 1.788 ± 0.1546 in control cells and 0.8746 ± 0.05338 in LD2-LD4 expressors) mean intensity ratio at FAs of U-118 MG-LD2-LD4 non-expressors (N=216 FAs from 15 cells) and expressors (N=190 FAs from 14 cells), using two-tailed unpaired t test. SEM is represented by error bars. ***: P value < 0.0001.

**Fig. S3.**

**
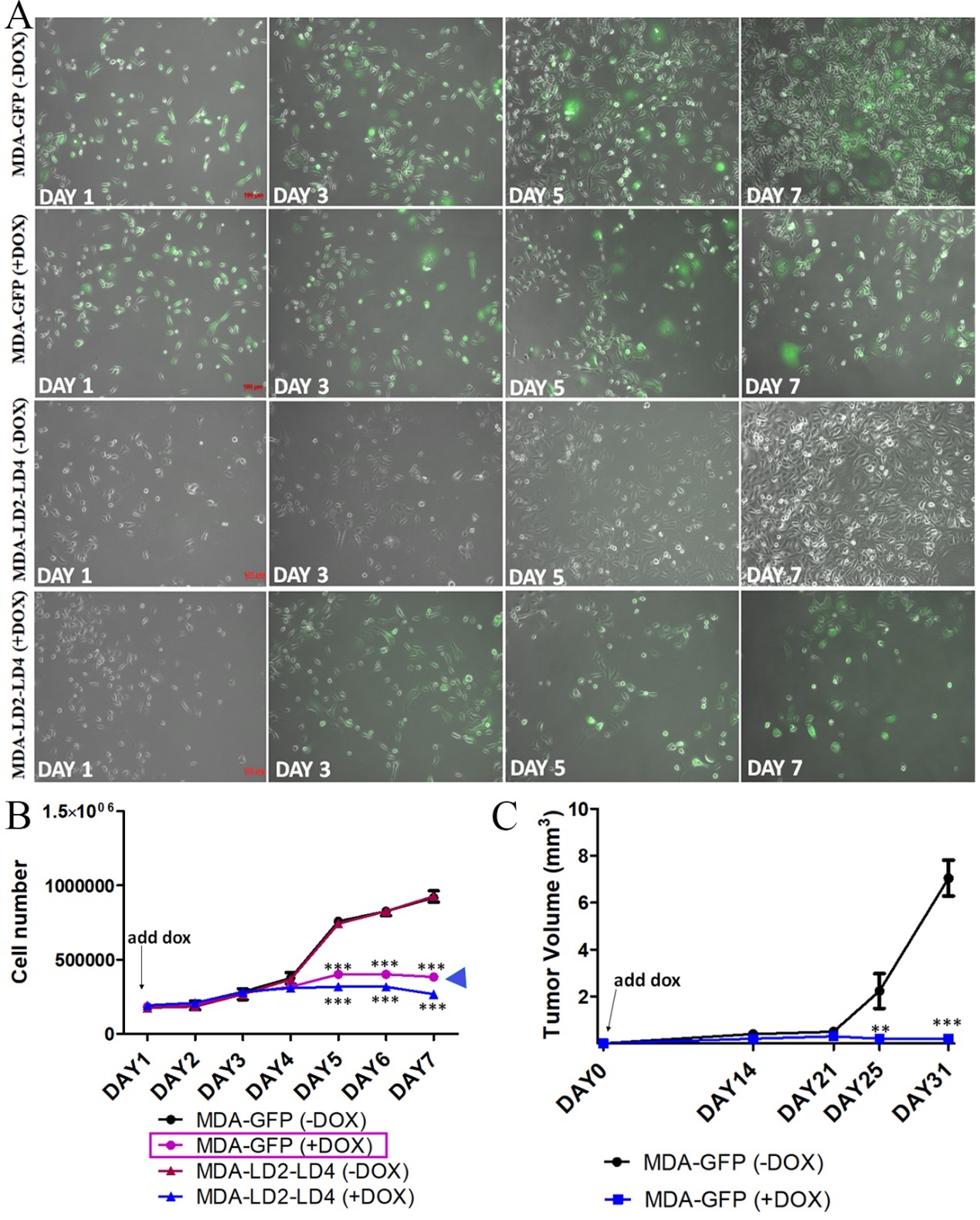
Fig. S3. Doxycycline has a strong anti-proliferative effect on MDA-MB-231 (MDA) cells. (A-B)** Representative fields and growth curve graph from three independent replicates of MDA-MB-231 (MDA) cells stably expressing GFP or inducibly expressing LD2-LD4. Doxycycline has a dramatic impact on MDA-GFP cell proliferation. Regular two-way ANOVA test (Bonferroni post-test) was used to compare MDA-GFP (-DOX) with (+DOX) cells and MDA-LD2-LD4 (-DOX) with (+DOX) cells. Scale bars: 100μm. SEM is represented by error bars. ***: P<0.001 **(C)** Tumor growth curve, following orthotopic injection of 1x10^6^ MDA-GFP cells in the mammary fat pad of NOD/SCID mice (N=5 mice each group). The curve shows inhibition of tumor growth upon doxycycline administration. Regular two-way ANOVA test (Bonferroni post-test) was used to compare MDA-GFP (-DOX) with (+DOX) mice. SEM is represented by error bars. **: P<0.01, ***: P<0.001.

**Fig. S4.**

**
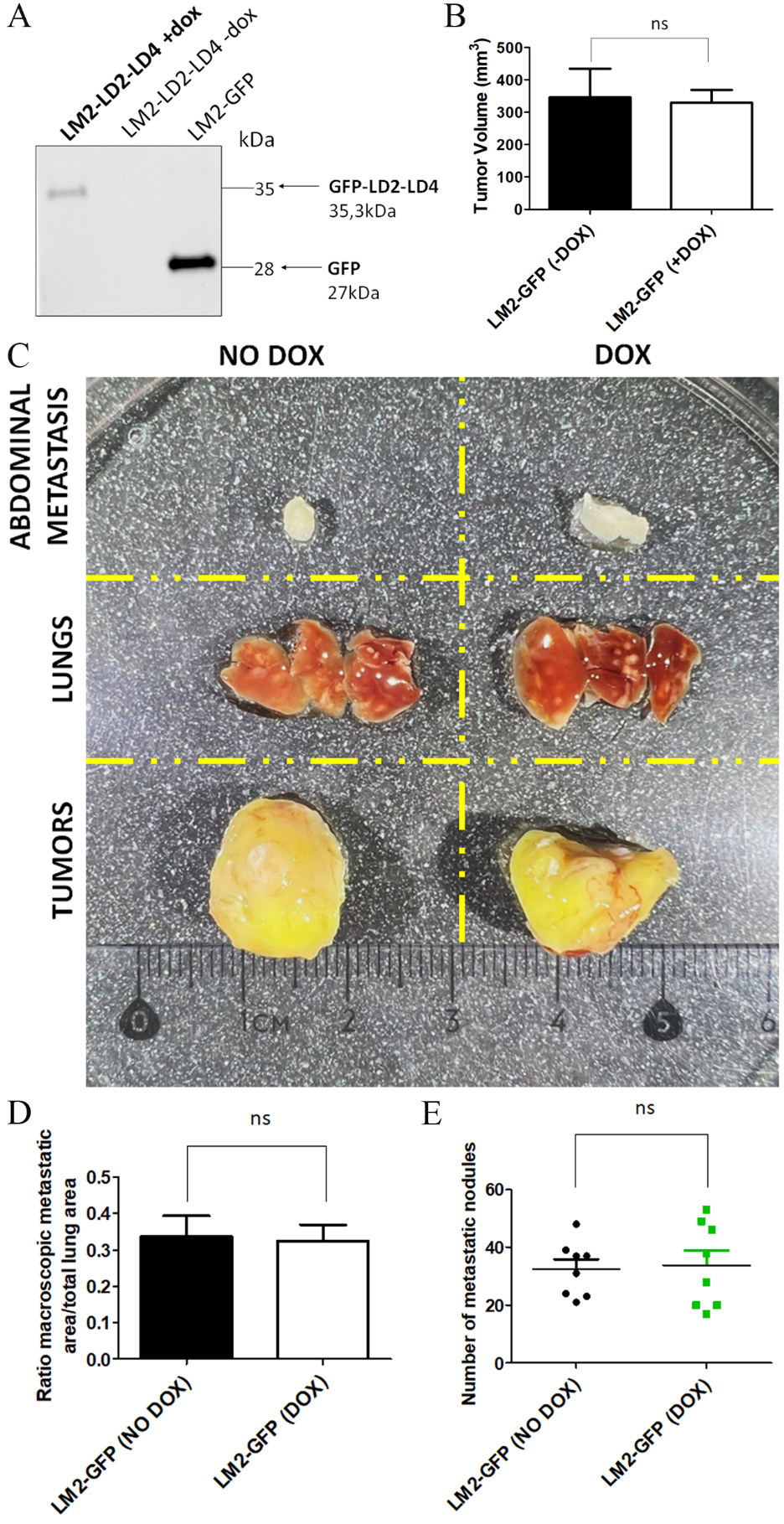
**

**Fig. S4. Doxycycline does not inhibit orthotopic MDA231-LM2-4175 tumor growth and metastatic spread.** **(A)** Western Blot showing GFP expression in MDA231-LM2-4175 (LM2) cells either stably expressing GFP-only or inducibly expressing LD2-LD4, using an anti-GFP antibody. **(B)** Quantification of excised tumor volume from -DOX and +DOX treated mice, using two-tailed unpaired t test. The mean tumor volume of non-treated mice (-DOX) is 345.9 ± 88.28, N=8 mice, and for the treated mice (+DOX) is 329.6 ± 39.34, N=8, ns: not significant. **(C)** Representative images showing tissues (tumors, lungs and abdominal metastatic foci) isolated from NOD/SCID mice injected with LM2-GFP cells. Mice from both GFP-groups (-DOX and + DOX) developed orthotopic tumors of comparable sizes and metastatic foci in lungs and abdominal region. **(D)** Quantification of macroscopic lung metastasis using two-tailed unpaired t test. The mean ratio of metastatic area/ total lung area of GFP non-treated mice (NO DOX) is 0.3369 ± 0.05724 (N=8 mice) and for GFP treated mice (DOX) is 0.3248 ± 0.04465 (N=8 mice). SEM is represented by error bars. ns: not significant. **(E)** Quantification of the number of lung metastatic nodules using two-tailed unpaired t test. The mean ratio of the number of lung metastatic nodules of GFP non-treated mice (NO DOX) is 32.50 ± 3.327 (N=8 mice) and for GFP treated mice (DOX) is 33.88 ± 5.111 (N=8 mice). SEM is represented by error bars. ns: not significant.

**Fig. S5.**

**
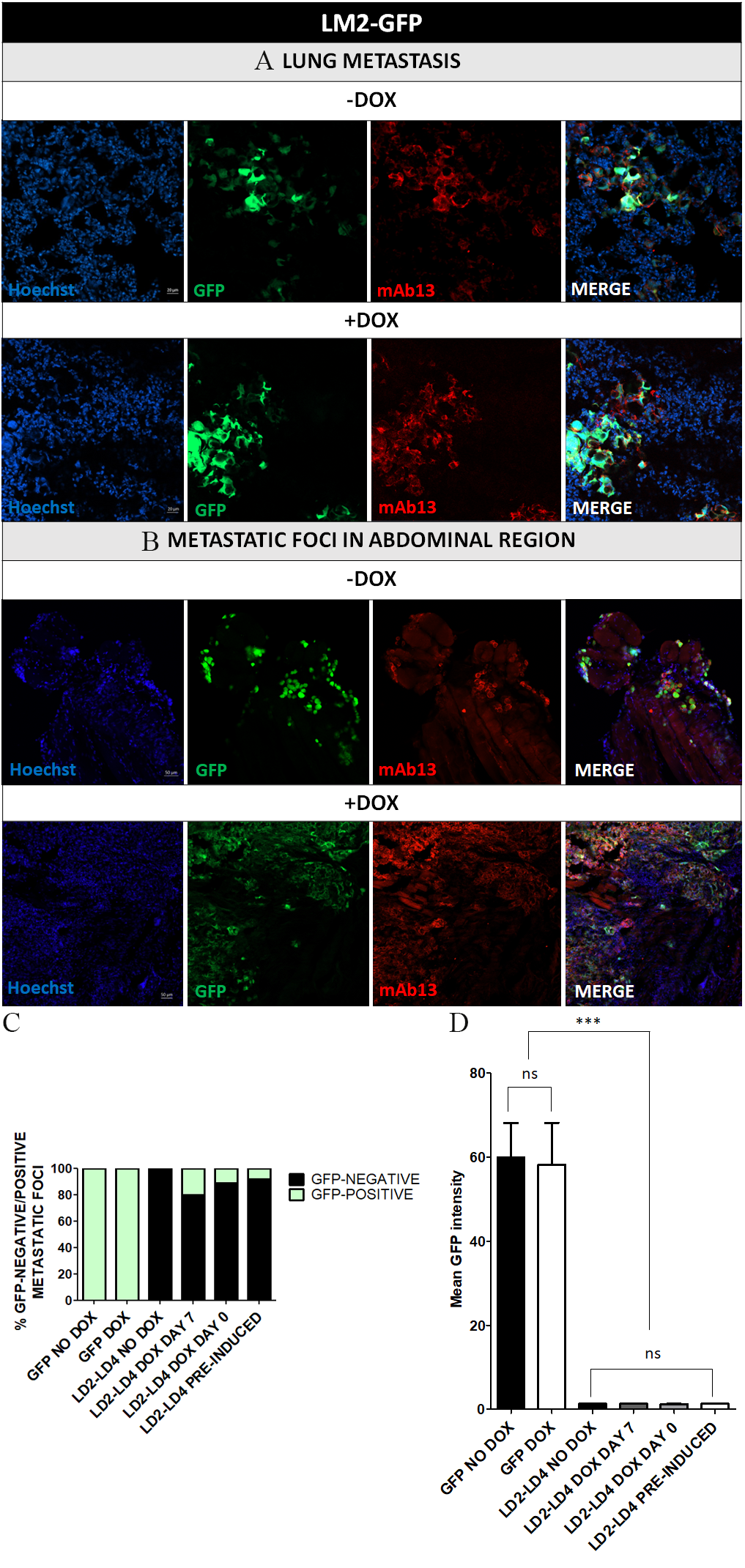
**

**Fig. S5. LM2-GFP cells detected in the lungs and abdominal region of non-treated and doxycycline-treated mice. (A-B)** Representative confocal images of frozen sections, processed for fluorescent immunohistochemical analysis. Human LM2-GFP cells were detected in the lungs **(A)** and abdominal region **(B)** in both doxycycline-treated and control mice, using an anti-human specific antibody, namely anti-integrin beta 1 (mAb13). Cell nuclei were visualized using Hoechst staining. Scale bars: 20μm **(A)**, 50μm **(B)**. **(C)** Quantification of the proportion of GFP-negative and GFP-positive lung metastatic foci of GFP- and LD2-LD4 mice groups. GFP-POSITIVE foci were those displaying 10% or higher GFP signal above the LD2-LD4 (NO DOX) group’s average GFP intensity level. For both GFP mice groups, 100% of metastatic foci were GFP-positive. For the LD2-LD4 (NO DOX) group, 100% of metastatic foci was GFP negative, for the LD2-LD4 (DOX DAY 7) group 80%, for LD2-LD4 (DOX DAY 0) group 88.89% and for LD2-LD4 (PRE-INDUCED) group 91.67%. **(D)** Quantification of mean GFP intensity of lung metastatic nodules of GFP- and LD2-LD4 groups, using one-way ANOVA and Tukey's Multiple Comparison Test. SEM is represented by error bars. ***: P<0.001. ns: not significant.

**Fig. S6.**

**
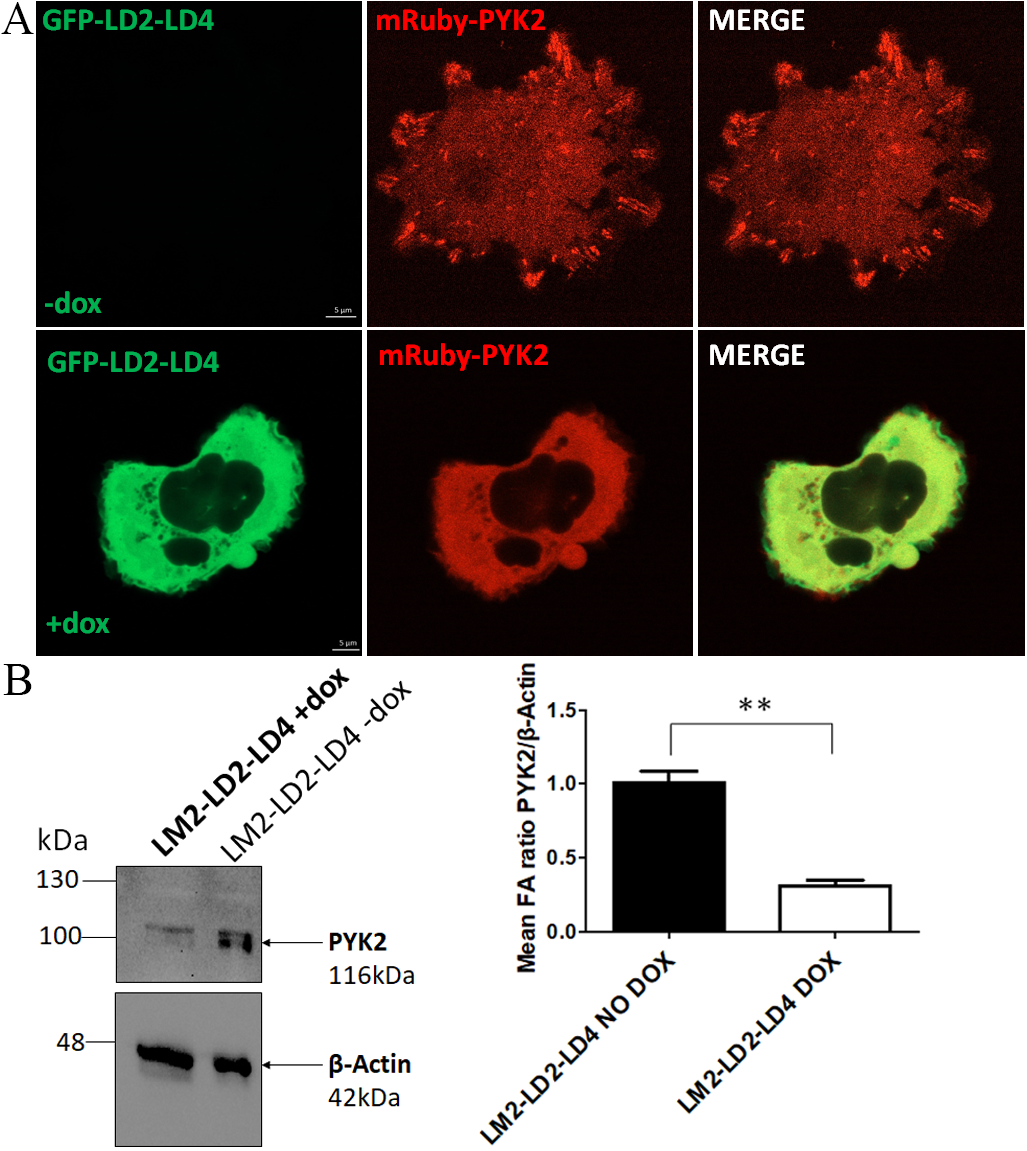
**

**Fig. S6. LD2-LD4 displaces PYK2 from focal adhesions. (A)** Live-cell imaging of LM2-LD2-LD4 cells (induced and uninduced) transiently transfected with mRuby-PYK2, showing displacement of PYK2 in +dox cells. **(B)** Representative Western blot from isolated FAs of inducible LM2-LD2-LD4 cells, blotted for PYK2 and β-actin. Quantification of mean FA ratio PYK2/β-Actin (1.010 ± 0.07638 in uninduced cells, 0.3100 ± 0.03786 in induced cells), shows reduction of PYK2 at the FAs of LM2 cells. N=3 independent experiments.

**Fig. S7.**

**
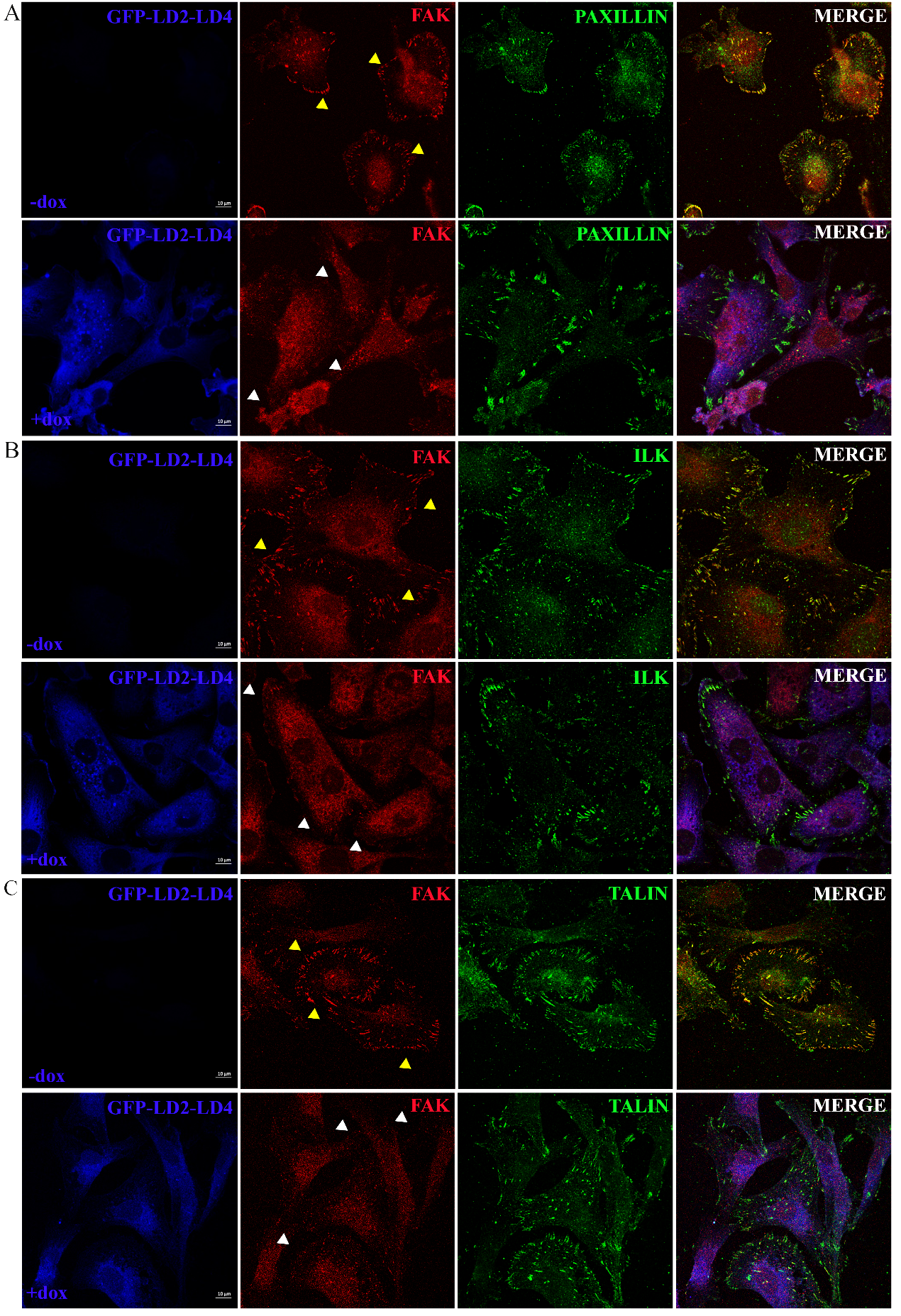
**

**Fig. S7. Displacement of FAK and PYK2 from FAs by LD2-LD4 expression does not affect overall FA composition.** Representative confocal images of LM2 cells inducibly expressing LD2-LD4, immunostained for FAK **(A-C)**, Paxillin **(Α)**, ILK **(Β)** and Talin **(C).** Yellow arrows are pointing FAs of uninduced cells and white arrows are pointing FAs of induced cells. LD2-LD4 effectively displaces FAK from FAs, without affecting overall FA composition. Scale bars: 10μm.

**Fig. S8.**


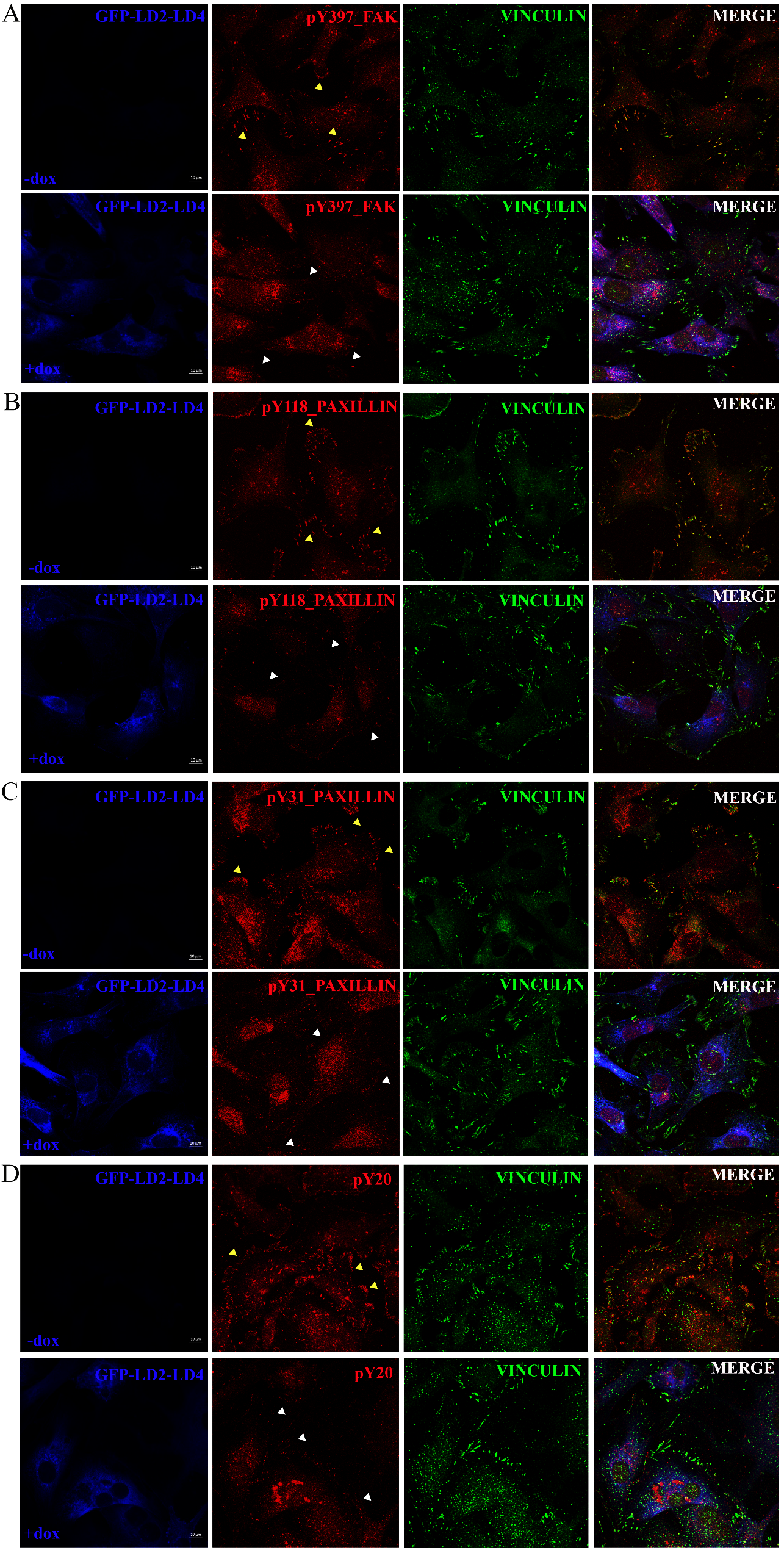


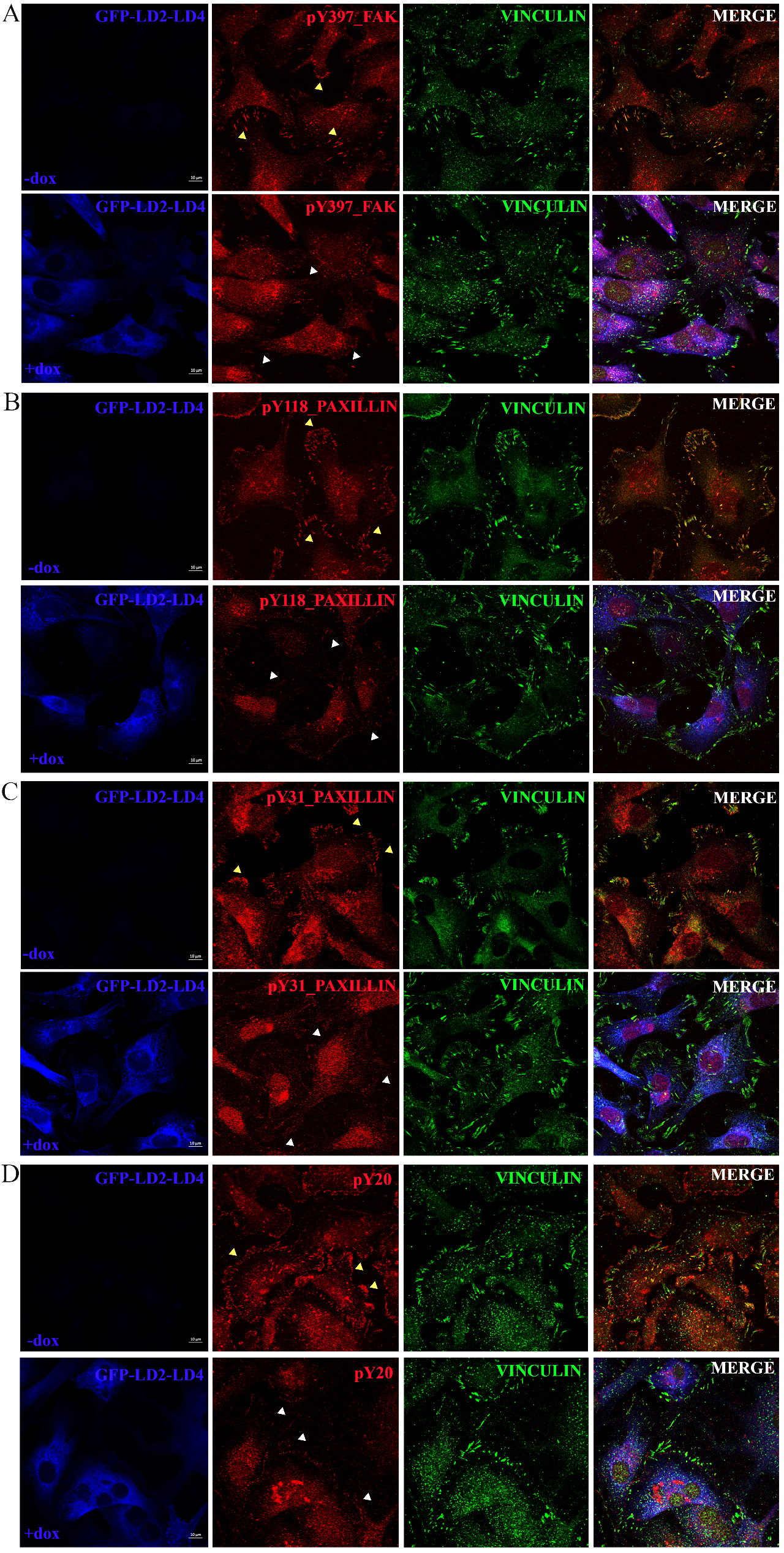


**Fig. S8. LD2-LD4 inhibits FAK kinase-dependent functions and downstream signaling in LM2 cells.** Representative confocal images of LM2 cells inducibly expressing LD2-LD4, immunostained for FAK Y397 **(A)**, paxillin Y118 **(B),** Paxillin Y31 **(C)**, and total tyrosine FA phosphorylation **(D).** Yellow arrows are pointing FAs of uninduced cells and white arrows are pointing FAs of induced cells. LD2-LD4 reduces FAK phosphorylation on tyrosine 397 **(A)**, as well as phosphorylation of downstream targets of FAK, such as Paxillin tyrosine Y118 **(B)** and Y31 **(C)** and total tyrosine FA phosphorylation **(D)** at FAs. Scale bars: 10μm.

**Fig. S9.**

**
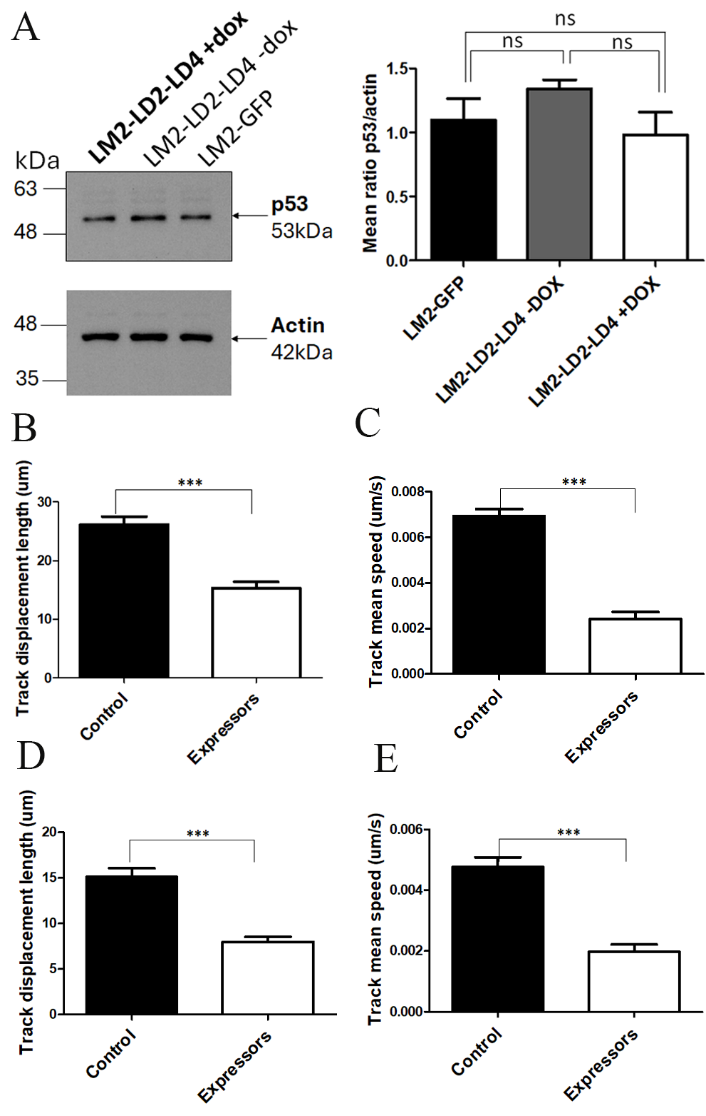
**

**Fig. S9. LD2-LD4 inhibits cell migration of SUM149 and SUM159 cells.** **(A)** Representative Western Blot and quantification, using LM2 cells stably expressing GFP or inducibly expressing LD2-LD4, show that LD2-LD4 expression does not affect p53 expression levels. Graph: Two-tailed unpaired t test was used for the quantification of total p53 levels over actin (mean ratio is 1.097 ± 0.1670 for GFP, 1.340 ± 0.0700 for uninduced and 1 0.9800 ± 0.1779 for LD2-LD4 expressing cells). N=3 independent experiments. **(B-C)** Quantification of mean track displacement length (μm) **(B)** and mean track speed (μm/sec) **(C)** of SUM149 cells transiently expressing GFP-LD2-LD4 or not (control), using two-tailed unpaired t test. The mean track displacement length of control cells is 26.12 ± 1.395 and for expressors is 15.33 ± 1.056. The mean track speed of control cells is 0.006944 ± 0.0002961 and for expressors is 0.002424 ± 0.0003019. N=252 control cells, 231 expressors, 3 independent experiments. SEM is represented by error bars. ***: P < 0.0001. **(C-D)** Quantification of mean track displacement length (μm) **(C)** and mean track speed (μm/sec) **(D)** of SUM159 cells transiently expressing GFP-LD2-LD4 or not (control), using two-tailed unpaired t test. The mean track displacement length of control cells is 15.11 ± 0.9611 and for expressors is 7.968 ± 0.5558. The mean track speed of control cells is 0.004764 ± 0.0003189 and for expressors is 0.001970 ± 0.0002430. N=254 control cells, 269 expressors, 3 independent experiments. SEM is represented by error bars. ***: P < 0.0001.

**Fig. S10.**

**
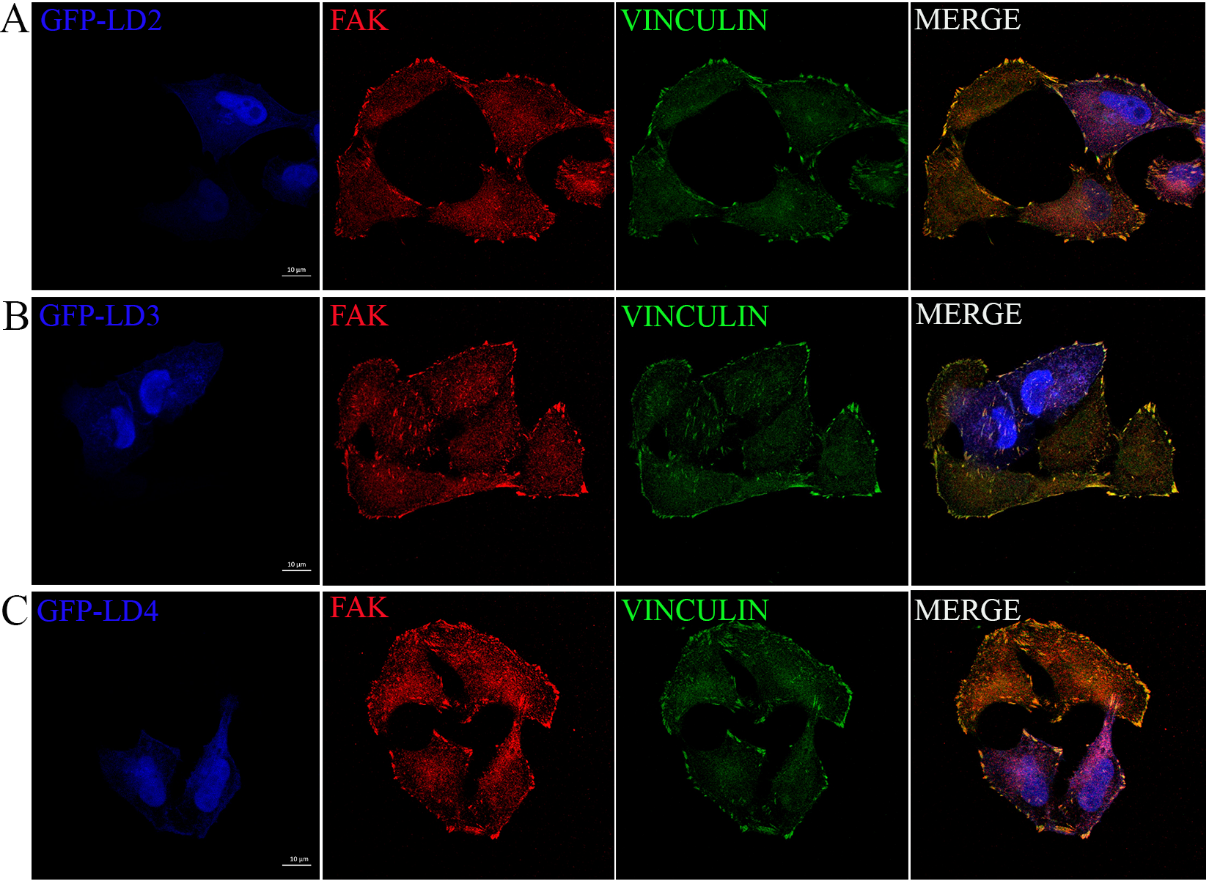
**

**Fig. S10. Single LD motifs do not displace FAK from focal adhesions.** Representative confocal images of HeLa cells transiently transfected with GFP-LD2 **(A)**, GFP-LD3 **(B)** and GFP-LD4 **(C)**, immunostained for FAK and Vinculin. Scale bars: 10μm.

**Fig. S11.**

**
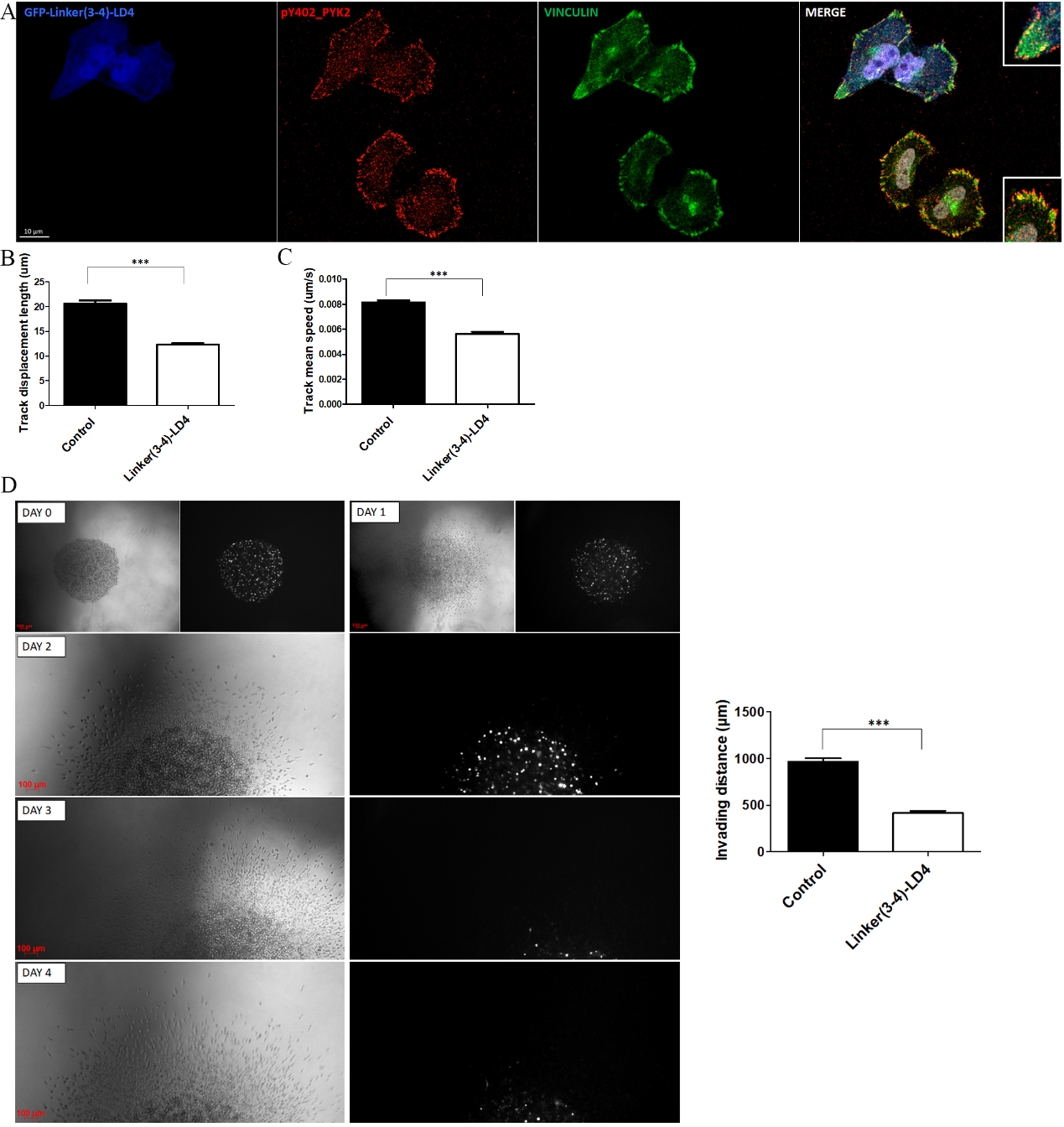
**

**Fig. S11. Linker(3-4)-LD4 peptide expression inhibits tumor cell migration and invasion. (A)** Representative confocal images of HeLa cells transiently transfected with GFP-Linker(3-4)-LD4, immunostained for pY402_PYK2 and Vinculin. Scale bar: 10μm. **(B-C)** Quantification of mean track displacement length (μm) **(B)** and mean track speed (μm/sec) **(C)** of MDA-MD-231 cells transiently expressing GFP-Linker(3-4)-LD4 or not (control), using two-tailed unpaired t test. The mean track displacement length of control cells is 20.62 ± 0.6146 and for expressors is 12.25 ± 0.3112. The mean track speed of control cells is 0.008131 ± 0.0001568 and for expressors is 0.005908 ± 0.0001883. N=749 control cells, 699 expressors, 3 independent experiments. SEM is represented by error bars. ***: P < 0.0001. **(D)** Images obtained on DAY0, DAY1, DAY2, DAY3 and DAY4, showing spheroids of LM2 cells (mixed population of untransfected control cells and cells transfected with Linker(3-4)-LD4). Control LM2 cells display increased invasion compared to LM2 expressors. Left panel: brightfield channel, right panel: GFP fluorescence channel. Graph: Quantification of invading distance, using two-tailed unpaired t test. The invading distance was calculated by measuring the distance between the representative leading invading cells from different areas of the setup and the core of the spheroid. The mean invading distance for control LM2 cells is 963.2 ± 38.03 and for expressors is 412.8 ± 24.53, N=6 spheroids per group. SEM is represented by error bars. ***: P < 0.0001.
